# ApoL6 associates with lipid droplets and disrupts Perilipin1-HSL interaction to inhibit lipolysis

**DOI:** 10.1038/s41467-023-44559-3

**Published:** 2024-01-02

**Authors:** Yuhui Wang, Hai P. Nguyen, Pengya Xue, Ying Xie, Danielle Yi, Frances Lin, Jennie Dinh, Jose A. Viscarra, Nnejiuwa U. Ibe, Robin E. Duncan, Hei S. Sul

**Affiliations:** 1 Department of Nutritional Sciences and Toxicology, University of California, Berkeley, Berkeley, CA 94720 USA; 2 Present Address: Department of Kinesiology and Health Sciences, University of Waterloo, Waterloo, ON N2T 2N4 Canada

**Keywords:** Obesity, Fat metabolism, Adipocytes

## Abstract

Adipose tissue stores triacylglycerol (TAG) in lipid droplets (LD) and release fatty acids upon lipolysis during energy shortage. We identify ApoL6 as a LD-associated protein mainly found in adipose tissue, specifically in adipocytes. ApoL6 expression is low during fasting but induced upon feeding. ApoL6 knockdown results in smaller LD with lower TAG content in adipocytes, while ApoL6 overexpression causes larger LD with higher TAG content. We show that the ApoL6 affects adipocytes through inhibition of lipolysis. While ApoL6, Perilipin 1 (Plin1), and HSL can form a complex on LD, C-terminal ApoL6 directly interacts with N-terminal Plin1 to prevent Plin1 binding to HSL, to inhibit lipolysis. Thus, ApoL6 ablation decreases white adipose tissue mass, protecting mice from diet-induced obesity, while ApoL6 overexpression in adipose brings obesity and insulin resistance, making ApoL6 a potential future target against obesity and diabetes.

## Abstract (web summary)

Lipolysis in adipose tissue releases fatty acids during fasting. Here, authors show that ApoL6, a lipid droplet-associated protein, is specifically expressed in adipocytes upon feeding to inhibit lipolysis by directly interacting with Perilipin 1 to competitively inhibit Perilipin 1-HSL interaction.

## Introduction

White adipose tissue (WAT) plays a central role in energy metabolism by storing excess energy as triglycerides (TAG). Adipocytes contain a large unilocular lipid droplet (LD) and are composed of core neutral lipids, TAG and some cholesterol ester, and surrounded by a single phospholipid monolayer. During periods of energy deprivation, hydrolysis of TAG stored in WAT is stimulated to release fatty acids (FA) into circulation so that other organs can use them as an energy source. Therefore, lipolysis within adipocytes represents a critical process in WAT’s function of FA release and needs to be regulated exquisitely according to nutritional conditions. In modern society, however, obesity characterized by increased adipose tissue mass has become an epidemic and is associated with chronic metabolic diseases, such as type 2 diabetes and insulin resistance. Furthermore, obesity-associated ectopic TAG storage in other tissues, such as liver, contributes to insulin resistance^1^.

Lipolysis proceeds in an orderly and regulated manner, by converting triacylglycerol (TAG) to diacylglycerol (DAG), monoacylglycerol (MAG) and FAs. These reactions are catalyzed by adipose triglyceride lipase (ATGL, PNPLA2, Desnutrin)^2–6^, hormone-sensitive lipase (HSL)^7^, and monoglyceride lipase (MGL)^8^, respectively. Recent advancement in understanding lipolysis has revealed various dynamic regulatory processes; Posttranslational modifications of lipases and LD-associated proteins by hormonal signals, such as catecholamines and insulin, have opposing effects on lipolysis. Phosphorylation of lipases and LD-associated proteins regulate assembly and disassembly of protein complexes for lipolysis on the surface of LD, thereby controlling lipases to access TAG and their catalytic activities^9–12^. In the fasted or stimulated state, hormones initiate signaling cascades that increase PKA activity to activate lipolytic pathways in adipocytes. For example, HSL is phosphorylated at S563 and S660 by PKA. ATGL is phosphorylated at S406 by AMPK, which has been shown to be activated by cAMP pathway^13–15^. In addition, PKA phosphorylates many components of the lipolytic complex. Perilipin 1 (Plin1, Perilipin A), a LD-associated protein, having six Protein Kinase A (PKA) consensus phosphorylation sites, R(R/K)XS, is phosphorylated by PKA^16^. In addition, a cofactor of ATGL, α/β hydrolase domain–containing protein 5, CGI-58 (ABHD5), is phosphorylated at S239 by PKA^17^. Phosphorylation initiates the translocation of lipases from the cytosol to LD, enabling protein-protein interaction to assemble the lipolytic complex on the Plin1 scaffold on LD^18,19^. Specifically, phosphorylation of Plin1 releases CGI-58 upon its phosphorylation. Then, the phosphorylated CGI-58 (P-CGI-58) interacts with phosphorylated ATGL for TAG hydrolysis^9,10,20^. Thus, ATGL hydrolytic activity increases by more than 20-fold upon interaction of ATGL with P-CGI-58. Phosphorylated HSL translocates from the cytosol to the surface of LD, where it binds to P-Plin1 to hydrolyze primarily DAG^21,22^. In fact, HSL translocation requires phosphorylation of both HSL and Plin1^7,23,24^. Finally, MGL cleaves the remaining fatty acid (FA) from the glycerol backbone^25^. In the fed state, insulin released from pancreatic islet β cells activates phosphodiesterase 3B to decrease cAMP levels and PKA activity, while insulin activating protein phosphatase 1 (PP-1)^26,27^ to potentially dephosphorylate target proteins, such as Plin1 and HSL, resulting in suppression of lipolysis in adipocytes^26,28^. Human and rodent genetic studies corroborate this concept of lipid mobilization pathway in adipocytes and the components of the lipolytic pathway may serve as therapeutic targets^29–31^. In addition, there are other LD-associated proteins that may regulate this lipolytic process. In this regard, G0/G1 switch gene 2 (G0S2) has been identified as an inhibitor of ATGL by binding with the catalytic patatin domain and suppressing activation^32^.

Here, in an attempt to better understand the role of LD-associated proteins on TAG metabolism of WAT, we identify an adipose-specific LD-associated protein, ApoL6. ApoL6 is a member of the ApoL family, sharing sequence identity within the amphipathic alpha-helix domain. We show that ApoL6 level in WAT is low in fasting but induced upon feeding. Genome-wide association studies (GWAS, https://www.gwascentral.org) identified two single nucleotide polymorphisms (SNPs) located at approximately 1 kb upstream of ApoL6 transcription, to associate with triglyceride and HDL cholesterol levels. ApoL6 ablation in mice causes a greatly diminished WAT mass with smaller adipocyte size. Conversely, overexpression of ApoL6 increases WAT mass with enlarged adipocytes with higher amount of TAG. We show that ApoL6 robustly inhibits lipolysis. ApoL6 directly interacts with the N-terminal domain of Plin1 to block Plin1-HSL interaction, thereby inhibiting lipolysis in adipocytes.

## Results

### ApoL6 is a LD-associated protein expressed primarily in adipose tissue

**Fig. 1:**
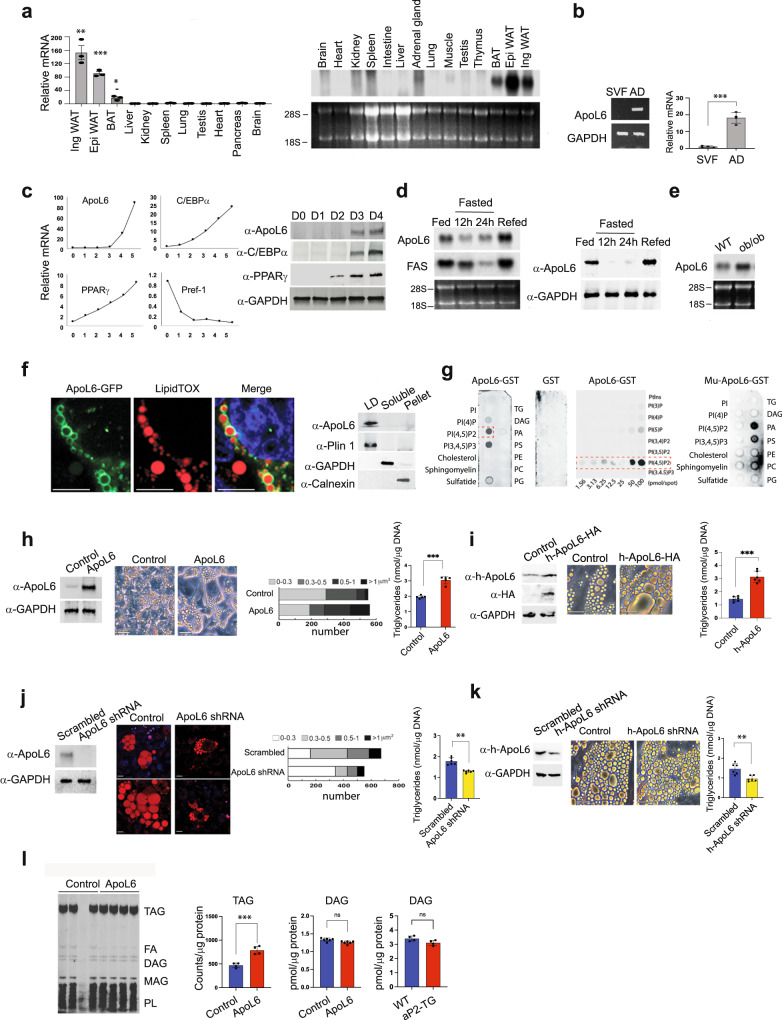
ApoL6 is a LD-associated protein and enhances triglyceride accumulation. **a** RT-qPCR and Northern blotting for ApoL6 mRNA levels in various tissues. (*n* = 3 mice, **p = 0.0061, **p = 0.0019, ***p < 0,001, two-tailed Student’s *t* test compared to expression levels in liver). Data are expressed as mean ± SD. **b** RT-PCR and RT-qPCR for ApoL6 mRNA levels in stromal vascular and adipocyte fractions of WAT (*n* = 4 mice, ***p < 0.001, two-tailed Student’s *t* test). Data are expressed as mean ± SD. **c** RT-qPCR (left) and immunoblotting (right) during 3T3-L1 adipocyte differentiation. **d** Northern blotting (left) and immunoblotting (right) of WAT. **e** Northern blotting of WAT. **f** Immunofluorescence of differentiated 3T3-L1 cells infected with ApoL6-GFP (green) adenovirus stained with (red), (bar = 10 μm) and immunoblotting after sucrose separation (right). **g** Immunoblotting after incubation of lipid membrane strips with either WT ApoL6-GST or control GST (right and middle) or mutated ApoL6 -GST (Mu-ApoL6-GST, aa286-292, from RYRKLQR to DYDDLQD, right). **h** Immunoblotting after infection of adenoviral ApoL6 in differentiated 3T3-L1 adipocytes, image (bar=10 μm), quantification of LD size and TAG levels (right, *n* = 5, ***p < 0.001, two-tailed Student’s *t* test). Data are expressed as mean ± SD. Experiment was repeated twice. **i** Human adipocytes differentiated from human fibroblasts were infected with lentiviral h-ApoL6-HA. Immunoblotting with h-ApoL6 antibody, cell images (bar = 10 μm), and TAG levels (*n* = 6, ***p < 0.001, two-tailed Student’s *t* test). **j** Immunoblotting after scramble and ApoL6-shRNA infection in 3T3-L1 adipocytes. LipidTOX staining (bar = 10 μm), quantification of LD sizes and TAG levels (*n* = 6. **p = 0.0025, two-tailed Student’s *t* test). Data represent mean ± SD. Independent experiments were repeated twice. **k** Human adipocytes were infected with lentiviral ApoL6 shRNA. Immunoblotting after infection, cell images (bar = 10 μm) and TAG levels (*n* = 6, **p = 0.003, two-tailed Student’s *t* test). **l** Lipids were extracted from [U-^14^C-TAG] pulse-labeled 3T3-L1 adipocytes and separated by TLC. Image for TLC plate. ^14^C-TAG was quantified by scintillation counting (*n* = 4, ***p < 0.001, two-tailed Student’s *t* test). DAG levels were measured in lipids extracted from 3T3-L1 adipocytes (*n* = 6, ns *p* = 0.0912, two-tailed Student’s *t* test) and WAT from mice (right, *n* = 4, ns *p* = 0.058, two-tailed Student’s *t* test). Data represent mean ± SD. Source data are provided as a Source Data file.

RT-qPCR and Northern blotting showed that ApoL6 mRNA levels were highly restricted to adipose tissues, including both iWAT and eWAT, also BAT although greatly lower (Fig. 1a). In adipose tissue, ApoL6 mRNA levels were mainly detected in the adipocyte fraction but not in the stromal vascular fraction (Fig. 1b). During 3T3-L1 adipocyte differentiation, as expected, we could detect decrease in preadipocyte marker Pref-1 and induction of PPARγ and C/EBPα during differentiation. ApoL6 expression was not detected in 3T3-L1 cells prior to adipocyte differentiation, but ApoL6 mRNA and protein levels were increased during adipocyte differentiation (Fig. 1c). We also tested human fibroblasts by differentiating them into adipocytes. Similar to that observed in murine 3T3-L1 cells, expression of ApoL6 was increased upon differentiation of human fibroblasts into adipocytes (Supplementary Fig. 1b).

We found that ApoL6 mRNA in adipose tissue of mice was decreased upon fasting but increased upon refeeding. In fact, ApoL6 protein in WAT was not detectable in fasting while greatly increased upon refeeding (Fig. 1d). ApoL6 mRNA expression was also higher in WAT of ob/ob mice than in non-obese C57BL6 mice (Fig. 1e). Overall, the pattern of ApoL6 expression was similar to those genes in lipogenesis that have SRE and E-box motifs where SREBP1c and USF, respectively, are known to bind to activate lipogenic genes upon feeding. Indeed, examination of promoter region of ApoL6 gene revealed several E-boxes motifs at −180, −488 and −605, as well as SRE motif at −373. Therefore, we constructed −1 kb ApoL6 promoter-luciferase plasmid and co-transfected with USF-1 or SREBP1c and found ApoL6 promoter activity to be significantly increased upon overexpression of USF-1 and SREBP1c (Supplementary Fig. 1c), indicating their involvement of the ApoL6 induction in the fed condition.

Examination of the amino acid sequence of ApoL6 indicated the presence of an apolipoprotein-like domain in the middle of ApoL6, a hydrophobic domain of potential membrane association near the N-terminus, and the coil-coil domain near the C-terminus. However, ApoL6 lacks a N-terminal signal sequence. To examine the intracellular localization of ApoL6, a full-length ApoL6-GFP fusion construct was transfected into differentiated 3T3-L1 adipocytes. We detected ApoL6-GFP fluorescence in adipocytes highly localized on LD stained by LipidTOX (Fig. 1f left). hApoL6 was also detected to be associated with LD (Supplementary Fig. 1d). We performed subcellular fractionation of the adipocyte lysates by centrifugation through sucrose gradient and subjected the fractions to immunoblotting. Similar to Plin1, ApoL6 was detected mainly in the floating LD fraction, but not in soluble fraction (cytosol) where GAPDH was detected, nor pellet fraction (all membranes and nucleus) where calnexin was detected (Fig. 1f right), confirming ApoL6 localization on LD.

Next, to understand how ApoL6 is associated with LD, composed of a TAG and CE core and a surface phospholipid monolayer, we incubated ApoL6-GST (Supplementary Fig. 1e) with membrane strip containing various phospholipid species. As shown in Fig. 1g, we detected a selective and strong ApoL6 binding to phosphorylated PIs, such as PI(4)P, PI(4, 5)P2 and PI(3,4,5)P3, but not other phospholipid species. GST control did not show any phospholipid binding. By using varying concentrations of phospholipid species, we found that ApoL6 preferably interacted with PI(4, 5)P2 (Fig. 1g middle).

Incubation with various ApoL6 deletion-GST fusion constructs showed that the C-terminal domain of ApoL6 (aa211-321) was responsible for binding to PI(4,5)P2 (Supplementary Fig. 1e). Lentivirus containing ApoL6 C-terminal domain (N-ApoL6) was used to infect differentiated 3T3-L1 cells. Immunofluorescence showed that N-ApoL6 was no longer associated with LD (Supplementary Fig. 1f). In fact, we found the consensus amino acid sequence for PIP2 binding, RKLQR (K/H/R)(K/H/R)XX (K/H/R) at aa288-292 of the ApoL6 C-terminal domain. Absence of ApoL6 binding to PI suggested that the positive charges might be important for ApoL6 interaction with PI(4,5)P2. Therefore, we generated ApoL6 constructs containing mutation of amino acids of positive charges, RYRKLQR, into amino acids of negative charges, DYDDLQD (Mu-ApoL6-GST). Indeed Mu-ApoL6-GST did not interact with PI(4,5)P2. However, we detected ApoL6 interaction mainly with phosphatidic acid (PA) (Fig. 1g right), These observations show the positive charges at the C-terminal domain of ApoL6 are critical for ApoL6 interaction with PI(4, 5)P2. We conclude that PI(4,5)P2, which is a minor yet important component of LD phospholipid monolayer, allows ApoL6 recruitment to LD, probably via electrostatic interaction.

### ApoL6 promotes triglyceride and LD accumulation in adipocytes

To understand the ApoL6 function in adipocytes, we overexpressed ApoL6 in 3T3-L1 cells by adenoviral infection (Fig. 1h left) and the cells were differentiated into adipocytes. Expression levels of adipogenic transcription factors, such as C/EBPα and PPARγ, as well as lipogenic enzymes, such as FAS and ACC, did not show differences between control and ApoL6 overexpressing cells, indicating neither adipogenesis nor lipogenesis was affected by ApoL6 overexpression (Supplementary Fig. 1g). Remarkably, however, we detected that LD were significantly larger in ApoL6 overexpressing adipocytes compared to control cells (Fig. 1h middle). Quantification of lipid area showed a higher lipid staining and content in ApoL6 overexpressing cells (Fig. 1h middle). Accordingly, total TAG content in ApoL6 overexpressing cells measured by a biochemical assay was significantly higher than control cells (Fig. 1h right). We also employed human adipocytes in documenting ApoL6 effect on TAG and LD accumulation. We infected adipocytes differentiated from human fibroblasts with h-ApoL6 lentivirus (h-ApoL6-HA). ApoL6 protein level was increased by 3-fold upon lentiviral infection (Fig. 1i left) and LD size was larger upon h-ApoL6 infection (Fig. 1i middle). Accordingly, TAG levels were 2.2-fold higher in h-ApoL6 overexpressing human adipocytes compared to control adipocytes (Fig. 1i right).

We next performed ApoL6 knockdown experiments in adipocytes. Infection of lentiviral ApoL6 shRNA greatly decreased ApoL6 expression in 3T3-L1 adipocytes (Fig. 1j left). shRNA mediated ApoL6 knockdown caused a significant decrease in LD size (Fig. 1j left and middle) and TAG content was decreased by 25% (Fig. 1j right). We also performed ApoL6 knockdown experiments in human adipocytes by infecting human shApoL6 lentivirus. ApoL6 expression was decreased by approximately 50% upon shApoL6 lentiviral infection. Similar to what was observed in 3T3-L1 adipocytes, the size of LD and TAG levels in human adipocytes were decreased also (Fig. 1k). Overall, these results demonstrate that ApoL6 can increase LD size and TAG content in adipocytes. We then separated the lipids by TLC to examine the changes in lipid metabolite levels. 3T3-L1 adipocytes were pulse-labeled with [U-^14^C] palmitic acid, then these cells were infected with control or ApoL6 virus in label-free media. Indeed, ApoL6 overexpressing cells had significantly higher ^14^C-TAG triglyceride levels by 60%, while showing noticeably lower FFA levels compared to control adipocytes. However, we did not detect accumulation of labeled DAG or MAG in ApoL6 overexpressing cells compared to control cells (Fig. 1l, Left). We also did not detect significant changes in DAG levels in WAT of mice upon ApoL6 overexpression (Fig. 1l, Right). Overall, these results indicate that ApoL6 overexpression leads to TAG accumulation and lowers FFA levels in adipocytes.

### ApoL6 inhibits lipolysis in adipocytes

**Fig. 2:**
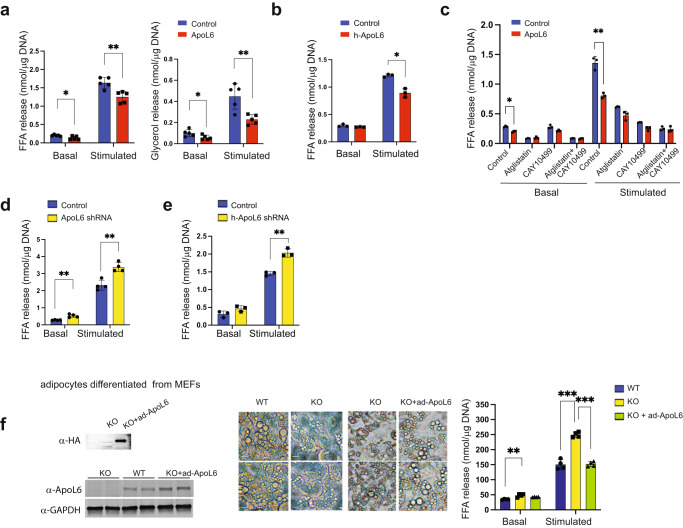
ApoL6 inhibits lipolysis. **a** Differentiated 3T3-L1 adipocytes were infected with inducible lentiviral ApoL6. Induction was achieved by treating the cells with Dox for 3 h before experiment. No Dox-treatment was used as control. FFA and glycerol release in the media from cells in basal and isoproterenol-stimulated conditions was measured. FFA (*n* = 5, *p = 0.016, **p = 0.0039, multiple *t* test), glycerol (*n* = 5, *p = 0.043, **p = 0.0055, multiple t test). Data represent mean ± SD. Independent experiment were repeat twice. **b** FFA release from human adipocytes after infection with lentiviral h-ApoL6-HA (*n* = 3, **p = 0.0027). Data represent mean ± SD. Experiment was repeated twice. **c** FFA release from differentiated 3T3-L1 cells cultured with Atglistatin (ATGL inhibitor) and CAY10499 (HSL inhibitor, *n* = 3, *p = 0.016, **p = 0.0013). Data represent mean ± SD. **d** FFA release from differentiated 3T3-L1 adipocytes after ApoL6 shRNA knockdown (*n* = 4, **p = 0.002 and 0.0019). **e** FFA release from human adipocytes after infection with lentiviral h-ApoL6 shRNA (*n* = 3, **p = 0.0016). Data represent mean ± SD. Experiment was repeated twice. **f** MEFs prepared from WT and ApoL6 KO E13.5 embryos were differentiated into adipocytes and infected with ApoL6-HA adenovirus. Immunoblotting with HA antibody and ApoL6 antibody (left), cell images (middle, bar = 10 μm) and FFA release (right, *n* = 4, **p = 0.0028, ***p < 0.001, two-way ANOVA test). Data represent mean ± SD. Independent experiments were repeated twice.

Thus far, we found that ApoL6 increased TAG and LD content in both rodent and human adipocytes, while not affecting adipocyte differentiation or lipogenic process. Also, we observe lower FFA levels in ApoL6 overexpressing adipocytes. Therefore, we hypothesized whether ApoL6 affects TAG hydrolysis, i.e., lipolysis. We examined lipolytic rate by measuring FFA and glycerol release in 3T3-L1 adipocytes after ApoL6 overexpression or knockdown. Indeed, FFA release was significantly lower in ApoL6 overexpressing adipocytes than in control cells in both basal and isoproterenol-stimulated conditions by approximately 30%. Glycerol release also was lower in basal and stimulated conditions by 30% and 60%, respectively (Fig. 2a). Next, we tested the effect of ApoL6 in human adipocytes. As observed in 3T3-L1 adipocytes, FFA release was lower significantly in h-ApoL6 overexpressing human adipocytes in stimulated conditions (Fig. 2b). These results reveal that overexpression of ApoL6 inhibits lipolysis in adipocytes.

When we treated 3T3-L1 adipocytes with ATGL inhibitor, Atglistatin, or HSL inhibitor, CAY10499, as expected, lipolysis was inhibited effectively. More importantly, after inhibitor treatment, we did not observe any differences in lipolysis between control and ApoL6 overexpressing cells (Fig. 2c). These results demonstrate that ApoL6 could not affect lipolysis when ATGL and HSL activities were inhibited. As indicated above, ApoL6 associates with LD by binding to PI(4,5)P2, although ApoL6 with PI(4,5)P2 binding site mutation may still associate with LD via binding to PA. Here, we examined whether ApoL6 binding to PI(4,5)P2 is critical for ApoL6 inhibition of lipolysis. However, overexpression of wild type (WT) ApoL6 and Mu-ApoL6 did not show any differences in the inhibition of lipolysis in both basal and stimulated conditions (Supplementary Fig. 2a). Although future studies on the significance of PI(4,5)P2 may be useful, our present results show that ApoL6 inhibition of lipolysis does not require PI(4,5)P2 binding by ApoL6.

Next, we performed ApoL6 knockdown to further examine ApoL6 effect on lipolysis. ApoL6 level was effectively decreased when we transduced ApoL6 shRNA lentivirus in differentiated 3T3-L1 adipocytes. ApoL6 knockdown resulted in a significant increase in lipolytic rate as measured by FFA release in both basal and stimulated conditions (Fig. 2d). Similarly, upon ApoL6 knockdown of human adipocytes, FFA release was higher in both basal and stimulated conditions, and differences in lipolysis were especially apparent upon isoproterenol treatment, increasing by 40% (Fig. 2e). To further verify the loss-of-function effect of ApoL6 on lipolysis, we employed adipocytes differentiated from fibroblasts (MEFs) isolated from ApoL6 KO mouse embryos. WT and ApoL6 KO adipocytes did not show any differences in the expression of adipogenic markers, such as C/EBPα and PPARγ, or lipogenic genes, such as FAS and mGPAT (Supplementary Fig. 2b). However, ApoL6 KO adipocytes had noticeably smaller LDs. Moreover, FFA release was significantly higher in ApoL6 KO adipocytes compared to WT adipocytes (Fig. 2f). Finally, we performed a rescue experiment using these adipocytes differentiated from ApoL6-KO MEFs. We infected the differentiated ApoL6 ablated adipocytes with ApoL6 adenovirus (Fig. 2f left). Indeed, adenoviral ApoL6 transduction into adipocytes differentiated from ApoL6-KO MEFs rescued lipid accumulation (Fig. 2f middle) and lowered FFA release (Fig. 2f right). All together, these results demonstrate that ApoL6 inhibits lipolysis to increase LD size in adipocytes.

### ApoL6 ablation leads to lower WAT mass with higher lipolysis in mice

**Fig. 3:**
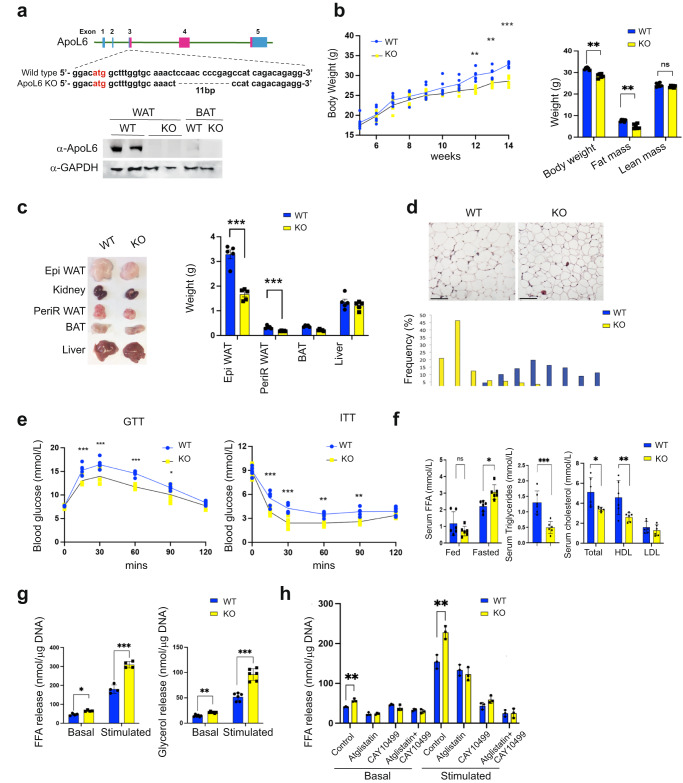
ApoL6 ablation in mice results in lean phenotype with increased WAT lipolysis. **a** 11 bp deletion in exon 3 of ApoL6 gene and ApoL6 protein level in WAT and BAT of WT and ApoL6 KO mice. **b** BW during HFD feeding (*n* = 6, **p = 0.0018 and 0.0072, ***p < 0.001, two-way AVOVA test) and EchoMRI after HFD (*n* = 6, ***p < 0.001, multiple *t* test). **c** Tissue pictures and tissue weights after 10 wks of HFD feeding (*n* = 5, ***p < 0.001). **d** H&E staining (bar = 100 μm) and quantification of cell sizes of WAT. **e** GTT and ITT after HFD (*n* = 6, GTT: ***p < 0.001, *p = 0.0347; ITT: **p = 0.0056 and 0.0013, ***p < 0.001, two-way ANOVA test). **f** Serum FFA in fed and fasted conditions (*n* = 6, *p = 0.011, multiple *t* test), TAG (*n* = 6, ***p < 0.001) and cholesterol levels in the fed condition (*n* = 6, *p = 0.0167, **p = 0.0058). Data represent mean ± SD. **g** FFA (*n* = 4, *p = 0.017, ***p < 0.001) and glycerol release (*n* = 6, **p = 0.005, ***p < 0.001) from dispersed adipocytes isolated from WAT. **h** FFA release from cultured primary adipocytes treated with inhibitors (*n* = 3, **p = 0.0028 and 0.0058). Data represent mean ± SD. Experiments were repeated twice.

To evaluate the physiological significance of adipose ApoL6 function in vivo, since ApoL6 was detected primarily in adipocytes of WAT, we generated global ApoL6 knockout mice (ApoL6 KO) by employing the CRISPR-Cas9 system. Guide RNA was designed to target the third exon of the ApoL6 gene (Fig. 3a upper). By sequencing of the ApoL6 genomic region, we verified one KO mouse line having an 11 bp deletion in the coding region of the third exon (Fig. 3a upper), which was germline transmitted (Fig. 3a upper). Immunoblotting showed absence of ApoL6 protein in WAT and BAT of ApoL6 KO mice (Fig. 3a lower), and we did not detect compensation from other ApoL family members (Supplementary Fig. 3a). We employed male WT and transgenic mice, unless specified otherwise. On normal chow diet, body weight did not show significant differences between WT and ApoL6 KO mice. We therefore subjected ApoL6-KO mice to HFD feeding. ApoL6 KO both male and female mice showed significantly lower body weights (BW) after HFD feeding for 7 wks (Fig. 3b left and Supplementary Fig. 3b left). EchoMRI showed ApoL6 KO mice having lower BW and WAT mass without changes in lean body mass (Fig. 3b right and Supplementary Fig. 3b right), while the food intake did not show difference between the KO and WT (Supplementary Fig. 3c). WAT depot weights also were significantly reduced in ApoL6 KO mice compared to WT mice (Fig. 3c), suggesting that ApoL6 KO mice were prevented from HFD-induced obesity. H&E staining and imaging, as well as quantification of WAT showed ApoL6 KO mice had smaller adipocyte size compared to WAT of WT mice (Fig. 3d). As expected, HFD feeding caused crown-like structures as well as an increase in inflammatory markers in WAT of WT mice, both of which decreased in ApoL6 KO mice (Supplementary Fig. 3e). Since adiposity affects glucose/insulin homeostasis, we performed glucose tolerance test (GTT) and insulin tolerance test (ITT). Indeed, after 10 wks of HFD feeding, WT mice had high glucose levels and insulin resistance as expected, whereas ApoL6 KO mice had significantly lower glucose levels at 15, 30 and 60 min after glucose injection (Fig. 3e). These ApoL6 KO mice also exhibited improved insulin sensitivity during ITT (Fig. 3e). Serum TAG and total cholesterol levels in the fed condition were significantly lower in ApoL6 KO mice than WT mice. Serum FFA levels did not show significant differences in the fed condition but were significantly higher in the fasted condition (Fig. 3f).

We next examined lipolysis by using dispersed adipocytes from WAT. Adipocytes from ApoL6 KO mice compared to WT mice showed significantly higher FFA and glycerol release (Fig. 3g and Supplementary Fig. 3d). In the basal condition, treatment with Atglistatin or both Atglistatin and CAY10499 inhibited FFA release in WT and ApoL6 KO adipocytes. In the simulated condition, treatment with Atglistatin, CAY10499, or both, also significantly decreased FFA release in WT by 14, 72 and 84%, respectively. These inhibitor treatments on ApoL6 KO adipocytes similarly inhibited lipolysis as in wild type cells (Fig. 3h). These results on lipolysis measured in dispersed adipocytes prepared from WAT of ApoL6 KO and WT mice are consistent with the above-described results obtained from cultured adipocytes, demonstrating that the absence of ApoL6 increases lipolysis in vivo to prevent diet-induced obesity.

### Adipocyte-specific ApoL6 overexpression increases WAT mass with fat accumulation in mice

**Fig. 4:**
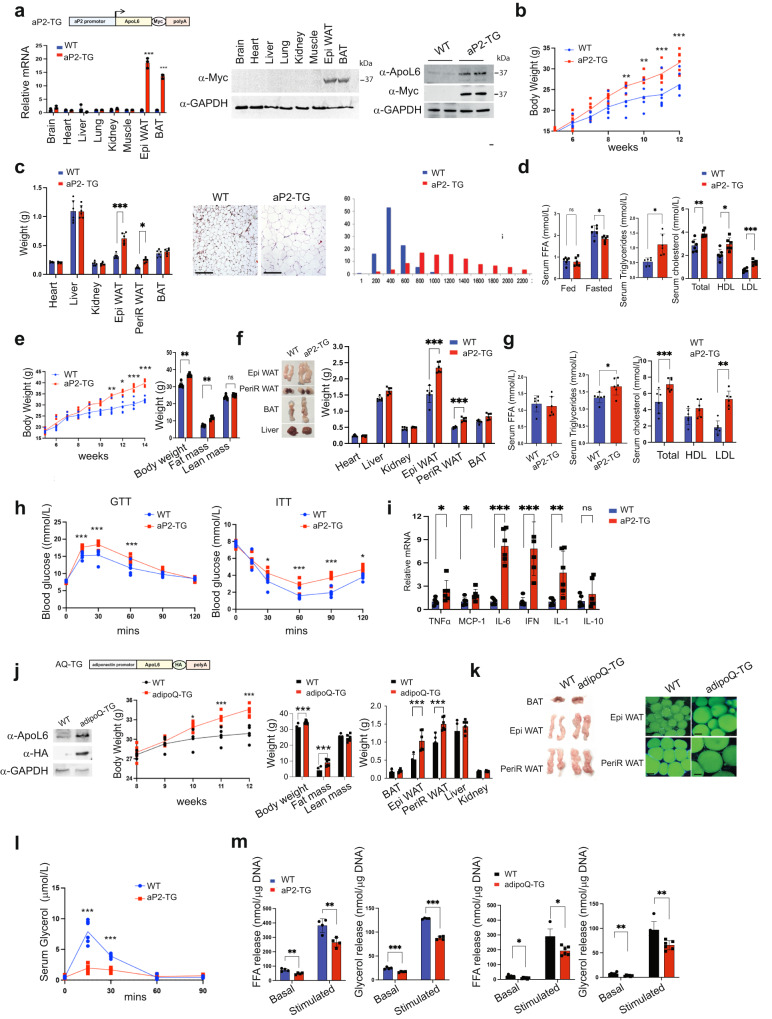
Overexpression of ApoL6 in adipocytes in mice increases WAT mass. **a** aP2-ApoL6 TG design (left upper), RT-qPCR for transgene expression (left lower, *n* = 3 mice, ***p < 0.001 compared to WT), immunoblotting of transgene expression in different tissues and ApoL6 protein levels in WAT (right). **b** BW in chow (*n* = 6 mice per group, **p = 0.0057 and 0.0027, ***p < 0.00, two-way ANOVA test). **c** Tissue weights of 24 wk-old mice (left, *n* = 6, *p = 0.03, ***p < 0.001, multiple *t* test). H&E staining (bar = 100 μm) and quantification of cell sizes of WAT (right two panels). **d** Serum FFA (fed and fasted, *n* = 6, *p = 0.0117, multiple *t* test), TAG (*n* = 6, *p = 0.0224), and cholesterol levels in fed condition (*n* = 6, **p = 0.0034, *p = 0.049). Data represent mean ± SD. **e** BW during HFD (*n* = 6, **p = 0.006, *p = 0.0104, ***p < 0.001) and EchoMRI after HFD (*n* = 6, **p = 0.007 and 0.005). **f** Tissue image and tissue weight after 12 wks of HFD feeding (*n* = 6, ***p < 0.001). Data represent mean ± SD. **g** Serum FFA, TAG (*n* = 6, *p = 0.0214) and cholesterol levels after HFD feeding in the fed condition (*n* = 6 ***p < 0.001, **p = 0.0239). **h** GTT (*n* = 6, ***p < 0.001) and ITT (*n* = 6, *p = 0.0162 and 0.0319, ***p < 0.001, two-way ANOVA test) after HFD, Experiment was repeated twice. **i** RT-qPCR for inflammatory markers in WAT after HFD (*n* = 6, *p = 0.045, 0.032, **p = 0.0089, ***p < 0.001, multiple *t* test). **j** adipoQ-ApoL6 TG design. Immunoblotting using lysates of WAT from adipoQ-ApoL6 TG. BW (*n* = 5 mice per group, ***p < 0.001) and WAT mass measured by using EchoMRI (*n* = 6, ***p < 0.001) and tissue weights after 12 wks on HFD (*n* = 6, ***p < 0.001). **k** Image of tissues and whole mount staining of WAT with LipidTOX green (bar = 10 μm) after HFD feeding. **l** In vivo lipolysis assay: Serum glycerol levels at different time points after mice were injected with isoproterenol at 10 mg/kg BW (*n* = 6, ***p < 0.001, two-way ANOVA test). Experiments were repeated twice. **m** FFA and glycerol release from dispersed adipocytes isolated from WAT of aP2-TG (*n* = 4, *p = 0.005 and 0.006, ***p < 0.001) (left two panel). FFA and glycerol release from dispersed adipocytes isolated from WAT of adipoQ-ApoL6 TG (*n* = 6. *p = 0.026, 0.021, **p = 0.0024 and 0.0023). Data represent mean ± SD. Experiments were repeated twice.

In studying ApoL6 function in adipose tissue in vivo, we next generated transgenic mice overexpressing ApoL6 in adipose tissue. We generated a transgenic mouse line overexpressing Myc-tagged ApoL6 driven by the −5.4 kb aP2 promoter (aP2-ApoL6 TG) (Fig. 4a–i). ApoL6 transgene expression was examined by RT-qPCR and immunoblotting (Fig. 4a and Supplementary Fig. 4a). ApoL6 transgene was expressed specifically in adipose tissue and was barely detectable in other tissues examined in 10-wk old mice (Fig. 4a). Immunoblotting using ApoL6 antibody showed that ApoL6 protein levels in aP2-ApoL6 TG WAT was approximately 3-fold higher than that of WT mice. When on normal chow diet, aP2-ApoL6 TG mice at 11–12 wks of age showed significantly higher body weights with higher WAT mass, in comparison to their WT littermates (Fig. 4b). Liver and other organ weights remained the same. H&E staining of WAT and quantification showed larger adipocyte sizes in WAT of aP2-ApoL6 TG, compared to WT mice (Fig. 4c). No histological differences were detected in BAT of WT and aP2-ApoL6 TG mice (Supplementary Fig. 4b). Gene expression of adipogenic transcription factors, such as C/EBPα and PPARγ, as well as lipogenic genes, such as FAS, ACC and mGPAT, did not show any differences between WT and aP2-ApoL6 TG mice (Supplementary Fig. 4c), indicating that ApoL6 did not affect adipogenesis or lipogenesis. The mice had comparable glucose and insulin tolerance on chow diet when subjected to GTT and ITT (Supplementary Fig. 4d). We then subjected aP2-ApoL6 TG mice to HFD. aP2-ApoL6 TG mice compared to WT littermates showed higher BW with higher fat mass starting after 7 wks of HFD feeding (Fig. 4e). There were no differences in food intake (Supplementary Fig. 4e). Female mice also showed similar increases in BW and fat mass (Supplementary Fig. 4f). aP2-ApoL6 TG mice had significantly higher WAT mass with larger adipocyte sizes compared to WT mice (Fig. 4f). Serum TAG, cholesterol and LDL/VLDL levels were elevated significantly in aP2-ApoL6 TG mice, although no differences in serum FFA levels were detected in the fed condition (Fig. 4g). GTT and ITT showed significantly higher glucose levels at various time points in aP2-ApoL6 TG compared to WT mice (Fig. 4h), indicating that aP2-ApoL6 TG mice on HFD were more glucose intolerant and insulin resistant than WT littermates. Expression of inflammatory markers increased in WAT of aP2-ApoL6 TG (Fig. 4i), indicating that elevated inflammation may have contributed to insulin resistance. Since insulin resistance is associated with hepatosteatosis, we also examine lipid accumulation in liver. Interestingly, in contrast to mice on chow diet that showed no histological differences, livers of aP2-ApoL6 TG compared to WT mice showed higher lipid accumulation by Oil Red O staining and by TAG content (Supplementary Fig. 4g). Therefore, we also examined ApoL6 expression in liver. ApoL6 was not detected in the livers of 12-wk old chow diet fed mice, but was detected in livers of HFD fed mice, indicating that HFD caused an induction of endogenous ApoL6 expression even in livers of mice when hepatic TAG content was high. We also found that ApoL6 expression was higher in livers of 30 wk-old than 12 wk-old mice (Supplementary Fig. 4h).

Since aP2 promoter is known to be not only active in adipocytes but may also be active in other cell types such as macrophages, we have also generated an additional ApoL6 transgenic mouse line overexpressing HA-tagged ApoL6 driven by the −5.2 kb adiponectin promoter (adipoQ-ApoL6 TG) (Fig. 4j, k). Similar to aP2-ApoL6 TG mice, ApoL6 protein levels in WAT of adipoQ-ApoL6 TG mice had approximately 3-4 folds higher ApoL6 protein levels than in WT mice. We examined these adipoQ-ApoL6 TG mice after subjecting them to HFD. The adipoQ-ApoL6 TG mice showed significantly higher BW after 10 wks. EchoMRI also showed adipoQ-ApoL6 TG mice having higher BW and WAT mass without changes in lean body mass (Fig. 4j). Weights of WAT depots, such as Epi-WAT and PeriR-WAT, but not weights of various other tissues were significantly higher in adipoQ-ApoL6 TG mice than WT mice after 12 wks of HFD (Fig. 4j left). Whole mount staining of WAT with LipidTOX green and quantification indicated that the sizes of adipocytes of adipoQ-ApoL6 TG mice were larger compared to WT mice (Fig. 4k right). GTT and ITT showed that these mice were glucose intolerant and somewhat insulin resistant, having increased expression of inflammatory markers in WAT (Supplementary Fig. 4j, k). We conclude that adipoQ-ApoL6 TG mice had similar phenotypes as aP2-ApoL6 TG mice, further confirming that the changes observed in aP2-ApoL6 TG mice and adipoQ-ApoL6 TG mice are indeed due to overexpression of ApoL6 in adipocytes. Overall, we conclude that ApoL6 inhibits lipolysis in adipose tissue that in turn increases adiposity and insulin resistance.

Next, to examine ApoL6 effect on lipolysis in vivo, we administered a nonselective β-agonist isoproterenol to artificially stimulate whole-body lipolysis^33^. We then examined blood glycerol concentration at different time points in WT and aP2-ApoL6 TG mice after isoproterenol administration. As expected, WT mice showed a rapid response of lipolysis to isoproterenol treatment, glycerol release increasing by 16- and 8-fold, 15 and 30 min after isoproterenol injection, respectively. More importantly, aP2-ApoL6 TG mice had a significantly lower increase in glycerol release of only 4-fold higher than basal level at 15 and 30 min after isoproterenol administration (Fig. 4l). These results clearly showed in an in vivo context a decrease in lipolysis from adipose tissue by ApoL6 overexpression. We also used dispersed adipocytes from WAT of these mice for lipolysis assay in vitro. We detected significantly lower FFA and glycerol release from dispersed adipocytes isolated from WAT of aP2-ApoL6 TG and adipoQ-ApoL6 TG mice than those from WT mice in both basal and stimulated conditions (Fig. 4m and Supplementary Fig. 4i). Overall, we conclude that ApoL6 inhibits lipolysis in adipose tissue.

### ApoL6 directly interacts with Plin1

**Fig. 5:**
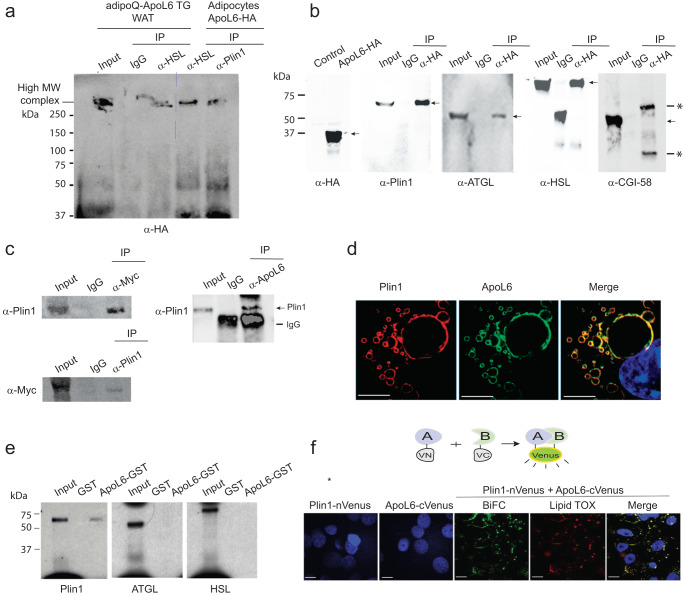
ApoL6 directly interacts with Plin1. **a** Total LD-associated proteins from WAT of adipoQ-ApoL6 TG mice and lysates of differentiated 3T3-L1 cells overexpressing ApoL6-HA were first IP with HA antibody, and the elution fractions were subjected to secondary IP with HSL antibody or Plin1 antibody. Immunoblotting with HA antibody after secondary IP using native gel. **b** Co-transfection of ApoL6-HA with Plin1, ATGL, HSL and CGI-58 in HEK293 cells. Immunoblotting after lysates were IP with HA antibody. Arrows indicate positions of interests; * indicates IgG band. **c** Immunoblotting after IP, using lysates of WAT of aP2-ApoL6 TG (left) and WT mice (right). **d** Immunofluorescence of differentiated 3T3-L1 adipocytes using Plin1 antibody (mouse) and ApoL6 antibody (rabbit) followed by Alexa fluor plus 594 anti-mouse (read) and Alexa Fluor plus 488 (green) anti-rabbit secondary antibodies (bar = 10 μm), respectively. **e** ApoL6-GST was incubated with S^35^ labeled in vitro transcribed/translated Plin1, ATGL and HSL before GST pull-down. **f** Bimolecular Fluorescence Complementation (BiFC) after transfection of Plin1 (fused to N-terminus of Venus) or ApoL6 (fused to C-terminus of Venus) alone (left two panels) and co-transfection of Plin1 and ApoL6 and merged with LipidTOX (red) staining in HEK293 cells (bar = 10 μm).

To start to understand the mode of action of ApoL6 in inhibiting lipolysis, we investigated ApoL6 interacting protein(s). We identified ApoL6 interacting proteins by immunoprecipitation (IP) with HA antibody using total LD-associated proteins extracted from the floating fraction of WAT of adipoQ-ApoL6 TG mice overexpressing HA-tagged ApoL6, as well as 3T3-L1 adipocytes overexpressing ApoL6-HA. The affinity purified proteins using HA antibody beads were subjected to mass spectrometry (MS) analysis and the experiments were repeated several times. Indeed, proteins involved in lipolysis, such as Plin1, HSL and MGL, were detected by MS analysis multiple times. Since ApoL6 is associated with LD in adipocytes and inhibits lipolysis, we hypothesized that ApoL6 might form a lipolytic complex on the LD surface. We tested our hypothesis by first immunoprecipitating with HA antibody for ApoL6, followed by IP with either HSL or Plin1 antibody. The samples were subjected to native acrylamide gel electrophoresis. Indeed, we detected the same very high molecular weight complex, when the samples of either purified by ApoL6 antibody followed by HSL antibody, or by ApoL6 antibody followed by Plin1 antibody (Fig. 5a). Potentially, a lipolytic complex containing ApoL6 may be formed on LD in hydrolyzing TAG in adipocytes. Although we did not pursue all components of this complex, MS analysis of this high molecular weight band detected several proteins, including ApoL6, Plin1 and HSL.

We next transfected into HEK293 cells with ApoL6-HA along with Plin1 and HSL, as well as other proteins that are known to be involved in lipolysis, such as ATGL and CGI-58. Co-IP experiments showed ApoL6 interaction with Plin1, HSL and ATGL, but not with CGI-58 (Fig. 5b). We could also detect ApoL6 interaction with Plin1 by using WAT. We performed IP with Myc antibody followed by immunoblotting with Plin1 antibody using total LD-associated proteins extracted from aP2-ApoL6 TG WAT overexpressing Myc-tagged ApoL6. Indeed, we detected ApoL6-Plin1 interaction. Conversely, IP with Plin1 antibody followed by immunoblotting with Myc antibody also detected ApoL6-Plin1 interaction (Fig. 5c left). Similar results were obtained when we used WT WAT by IP with ApoL6 antibody for ApoL6 and immunoblotting with Plin1 antibody to detect interaction of endogenous ApoL6 and Plin1 (Fig. 5c right). We also detected hApoL6 interaction with Plin1 in human adipocytes (Supplementary Fig. 5A). We then performed immunofluorescence experiment for localization. Immunofluorescence using ApoL6 and Plin1 antibodies along with fluorescence conjugated secondary antibodies showed co-localization of these proteins on the LD surface in differentiated 3T3-L1 adipocytes (Fig. 5d), as well as in WAT sections (Supplementary Fig. 5b).

While ApoL6 may be present in a large complex composed of lipases and other LD-associated proteins for lipolysis, our next goal was to identify a specific protein that directly interacts with ApoL6. To this end, we performed GST pull-down assay. We incubated ApoL6-GST with [^35^S]-Methionine labeled in vitro translated Plin1, HSL, or ATGL. Indeed, ApoL6 did not show direct interaction with either HSL or ATGL. In contrast, ApoL6 showed a signal when incubated with in vitro translated Plin1, indicating direct interaction of ApoL6 with Plin1, but not with HSL or ATGL (Fig. 5e). We did not detect ApoL6-ATGL direct interaction when we used purified ATGL in the GST pull-down assay (Supplementary Fig. 5c). Next, we employed Bimolecular Fluorescence Complementation (BiFC) assay. We generated expression vectors of ApoL6 fused to the C-terminus of Venus protein and Plin1 fused to the N-terminus of Venus. As expected, no signal was detected when Plin1-nVenus or ApoL6-cVenus were transfected individually (Fig. 5f). When both were transfected together, BiFC showed a signal that merged with LipidTOX staining, demonstrating the BiFC signal localized on the LD. These results further show ApoL6 interaction with Plin1.

### C-terminal domain of ApoL6 is critical for Plin1 interaction and for inhibition of lipolysis

**Fig. 6:**
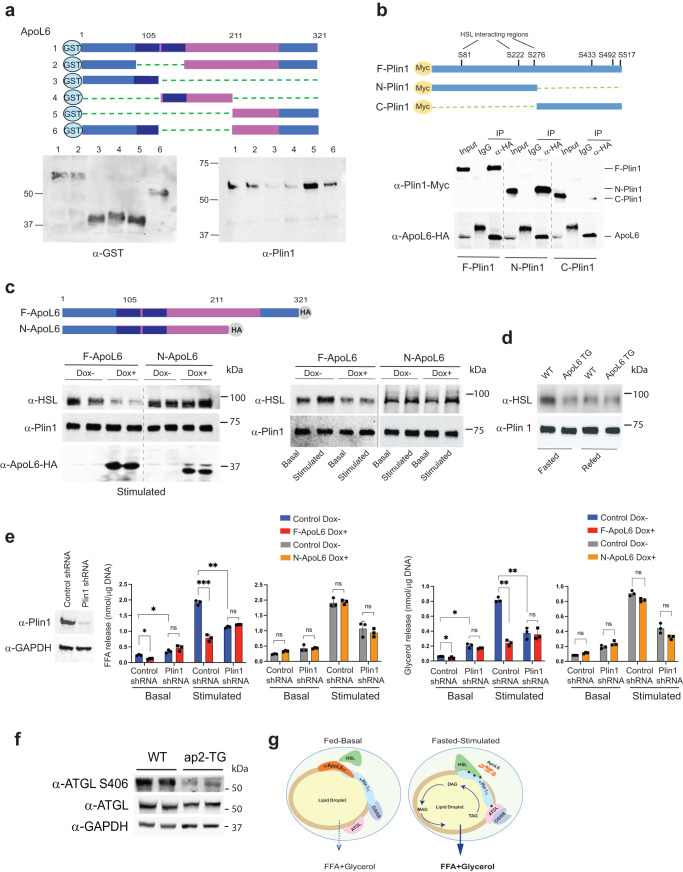
C-terminal domain of ApoL6 is critical for Plin1 interaction to inhibit lipolysis. **a** ApoL6-GST deletion constructs (upper), immunoblotting after GST purification (lower left). Purified GST proteins then were incubated with purified full length Plin1. Immunoblotting with Plin1 antibody after GST pull-down (lower right). **b** Myc-tagged Plin1 deletion constructs (upper) were co-transfected with F-ApoL6-HA into HEK293 cells. Immunoblotting with Myc antibody after IP with HA antibody. **c** Inducible lentiviral F-ApoL6-HA and C-terminal deletion ApoL6 HA (N-ApoL6) (upper) were used to infect HEK293 cells after co-transfection with Plin1 and HSL. Immunoblotting with HSL antibody after IP with Plin1 antibody (left). Immunoblotting with HSL antibody after IP with Plin1 antibody in differentiated 3T3-L1 adipocytes induced to overexpress F-ApoL6-HA and N-ApoL6-HA (right). **d** Immunoblotting with HSL antibody after IP with Plin1 antibody using total LD-associated proteins of WAT from wild type and aP2-ApoL6 TG mice in fasted and fed conditions. **e** Immunoblotting after transduction of lentiviral Plin1 shRNA in differentiated 3T3-L1 adipocytes. FFA and glycerol release from 3T3-L1 adipocytes in basal and stimulated conditions (FFA: *n* = 3, *p = 0.019 and 0.038, **p = 0.0052, ***p < 0.001; glycerol: *n* = 3, *p = 0.046, 0.012, **p = 0.0011 and 0.0016, two-way ANOVA test). Data represent mean ± SD. Experiments were repeated twice. **f** Immunoblotting of lysates from WAT of fasted WT and aP2-ApoL6 TG mice after HFD. **g** Schematic model of lipolysis in adipocytes in fed-basal condition and in fasted-stimulated condition.

To examine the domains of ApoL6 and Plin1 that interact, we generated and purified various ApoL6-GST deletion proteins (Fig. 6a top). The same ApoL6-GST deletion proteins used for incubation with phospholipid membrane strips in Fig. 1 were incubated with purified Plin1 (Fig. 6a lower left). The GST pull-down assay showed that all ApoL6-GST deletion proteins showed interaction with Plin1 except those containing deletion of 1/3 of ApoL6 C-terminal domain (constructs #3 and #4), indicating ApoL6 C-terminal domain of aa211-321 is required for interaction with Plin1 (Fig. 6a lower right). Next, to identify which domain of Plin1 is responsible for ApoL6 interaction, we generated two Plin1 deletion constructs, N-Plin1 (aa1-280) containing HSL binding domain and C-Plin1 (aa281-517) containing ATGL and CGI-58 binding domain (Fig. 6b). The full-length Plin1 (F-Plin1) and its deletion constructs along with full-length ApoL6-HA were overexpressed in HEK293 cells. IP with HA antibody followed by immunoblotting with Plin1 antibody showed that ApoL6 interacted with F-Plin1 or N-Plin1, but not with C-Plin1 (Fig. 6b). Overall, we conclude that the C-terminal ApoL6 interacts with the N-terminal Plin1.

It is critical for the stimulation of lipolysis that Plin1 interacts with HSL to allow HSL translocation from the cytosol to LD. To test whether ApoL6-Plin1 interaction has any impact on Plin1-HSL interaction, along with Plin1, we infected lentivirus containing either full-length ApoL6 (F-ApoL6-HA) or ApoL6 deleted of C-terminal domain (aa1-210, N-ApoL6-HA) in a doxycycline (Dox) inducible manner into HEK 293 cells. In the stimulated condition, overexpression of F-ApoL6-HA by Dox treatment compared to control, non-Dox treated cells significantly diminished Plin1-HSL interaction (Fig. 6c left), signifying that ApoL6 interaction with Plin1, in turn, interrupts Plin1-HSL interaction. In cells infected with ApoL6-N-HA lentivirus, however, no differences in Plin1-HSL interaction were observed whether the cells were overexpressing N-ApoL6 or not (Fig. 6c left), demonstrating that ApoL6 C-terminal domain (aa211-321) interaction with Plin1 is required for disruption of Plin1-HSL interaction. We also tested effect of ApoL6 on interaction of endogenous Plin1 and HSL in adipocytes. The 3T3-L1 adipocytes infected with inducible F-ApoL6-HA, were immunoprecipitated with Plin1 followed by immunoblotting with HSL antibody. In non-Dox treated control cells, as expected, Plin1-HSL interaction was stronger in the stimulated compared to basal condition. In contrast, overexpression of F-ApoL6-HA decreased the Plin1-HSL interaction. Moreover, cells infected with Dox-inducible N-ApoL6-HA, showed no differences whether cells were treated with Dox or not, in either basal or stimulated conditions (Fig. 6c right). We next used total LD-associated proteins from WAT to test interaction of endogenous proteins. Upon IP with Plin1, strong Plin1-HSL signal showing their interaction was detected from WAT of fasted compared to fed WT mice. However, Plin1-HSL interaction was diminished in WAT of either fasted or fed ApoL6 TG mice (Fig. 6d). Taken together, we conclude that, via its C-terminal domain, ApoL6 interacts with Plin1, and the ApoL6-Plin1 interaction prevents Plin1-HSL interaction.

It has been reported that Plin1 phosphorylation at S-81, S-222 and S-276 are required for HSL interaction with Plin1 on LD surface under the stimulated condition^34^. To test whether Plin1 phosphorylation affects ApoL6 interaction with Plin1, we generated Plin1 mutation construct by replacing those three serine residues to aspartate (Plin1SD) to mimic the phosphorylated form. We incubated ApoL6-GST with either WT Plin1 or Plin1SD that were purified from 293 FT cells transfected with those expression constructs. Immunoblotting showed that ApoL6-Plin1 interaction was not altered (Supplementary Fig. 6a). We also co-transfected HA tagged ApoL6 with either Myc-tagged WT Plin1 or Myc-tagged Plin1SD into 293 FT cells and performed IP with Myc antibody. Immunoblotting with HA antibody showed that ApoL6-Plin1 interaction was not altered when Plin1SD compared to WT Plin1 was used (Supplementary Fig. 6b). Next, we tested whether ApoL6 can inhibit HSL binding to Plin1SD under basal condition. We transfected 293 FT cells with ApoL6 and HSL, along with Myc-tagged Plin1 or Myc-tagged Plin1SD (Supplementary Fig. 6c left). IP with Myc antibody followed by immunoblotting with HSL antibody showed that HSL interaction with WT Plin1 and Plin1SD were not different, and ApoL6 prevented Plin1-HSL interaction in either case (Supplementary Fig. 6c right). These results indicate that ApoL6 prevents Plin1-HSL interaction regardless of the phosphorylation status of Plin1. In differentiated 3T3-L1 adipocytes, Plin1SD overexpression resulted in increased lipolysis in the basal condition, but it did not affect inhibitory effect of ApoL6 in these cells (Supplementary Fig. 6d).

While Plin1 has been reported to inhibit basal lipolysis^35,36^, Plin1 has also been reported to be required for stimulated lipolysis^11^. For stimulated lipolysis, HSL is phosphorylated and translocated to the LD and binds to phosphorylated Plin1 for lipolytic action^19^. If ApoL6 prevents Plin1-HSL interaction, ApoL6 inhibition of lipolysis should require the presence of Plin1. In testing whether Plin1-HSL interaction is required for ApoL6 inhibition of lipolysis, we performed Plin1 knockdown in 3T3-L1 adipocytes by transducing lentiviral Plin1 shRNA (Fig. 6e). We then induced expression of ApoL6 before measuring lipolytic rate. In the basal condition, consistent with previous reports, Plin1 knockdown significantly increased FFA and glycerol release by 1.6 and 2.3-fold, respectively. More importantly, ApoL6 overexpression decreased FFA and glycerol release by 55% and 41%, respectively. This indicates that the inhibitory effect of ApoL6 in the basal condition was diminished upon Plin1 knockdown. In the stimulated condition, as expected, FFA and glycerol release was lower by 43% and 54%, respectively, in Plin1 knockdown cells compared to control cells. Moreover, although ApoL6 inhibited lipolysis by more than 70% in control cells, ApoL6 could not further inhibit lipolysis in Plin1 knockdown cells. Overall, these data indicate that the inhibitory effect of ApoL6 on lipolysis requires the presence of Plin1. To better document the role of ApoL6-Plin1 interaction in lipolysis, we overexpressed N-ApoL6 which does not contain the Plin1 interacting domain. Indeed, N-ApoL6 overexpression no longer lowered FFA and glycerol release in both basal and stimulated conditions, regardless of presence of Plin1 (Fig. 6e). These results further confirm that the C-terminal domain of ApoL6 is required for its interaction with Plin1, which prevents Plin1-HSL interaction, thereby resulting in inhibition of lipolysis. Interestingly, however, we detected TAG, but not DAG, accumulation upon ApoL6 overexpression. This could have been due to lower ATGL S406 phosphorylation we detected in WAT of ApoL6-TG mice (Fig. 6f). In this regard, we previously reported S406 phospholyation of ATGL in activating lipolysis^13^.

In conclusion, as depicted in the graphical abstract (Fig. 6g), in the fed state, i.e., non-stimulated basal condition, ApoL6 expression is induced and, the C-terminal domain of ApoL6 interacts with N-terminal domain of Plin1, preventing HSL docking to Plin1. In the fasted state, i.e., stimulated condition, decreased ApoL6 levels allow Plin1 to interact with HSL to stimulate lipolysis.

## Discussion

Here, we identify a new LD-associated protein ApoL6, which is highly restricted to adipose tissue and is induced in the fed compared to fasted state to control adipose lipolysis. ApoL6 inhibits lipolysis by directly interacting with Plin1. Thus, ApoL6 knockdown or ablation results in smaller LD with lower TAG content in adipocytes and in WAT of mice that become resistant to diet-induced obesity. Conversely, overexpression of ApoL6 in adipocytes leads to larger LD with high TAG content in adipocytes and in WAT of mice with greater WAT mass, making mice more susceptible to weight gain, resulting in impaired glucose tolerance, insulin resistance, and ectopic TAG accumulation in the liver.

### ApoL6-Plin1 interaction

We detected various proteins involved in lipolysis, including Plin1, HSL, and MGL as a large ApoL6-containing protein complex, a so-called lipolytic complex. Although ApoL6 interacting proteins form a large complex, ApoL6 can directly bind only to Plin1. We also revealed that ApoL6 C-terminal domain is required for the ApoL6-Plin1 binding and that ApoL6 interacts with the N-terminal domain of Plin1. In this regard, ApoL6 contains a coiled-coil domain at its C-terminal region proposed to act as a molecular spacer that can either separate functional domains or scaffold large macromolecular complexes^37^. In fact, Plin1 is the first and best-studied LD-associated protein^38,39^. In the stimulated state, Plin1 is phosphorylated to release phosphorylated CGI-58. Then, the Plin1 N-terminal domain with 3 phosphorylation sites becomes a docking site for phosphorylated HSL to access LD^40^. We claim that ApoL6 interacts with the N-terminal domain of Plin1 in order to compete with HSL interaction with Plin1, thereby keeping HSL in a “stand by” status. In this regard, it has been shown that phosphorylation of several specific serine resides at the Plin1 N-terminal domain is required for Plin1-HSL interaction. However, we found that the ApoL6-Plin1 interaction occurs regardless of Plin1 phosphorylation status, preventing HSL-Plin1 interaction. Moreover, we found that ApoL6 C-terminal domain is critical for the ApoL6-Plin1 interaction and for ApoL6 binding to PI(4,5)P2 to associate with LD. However, mutation of ApoL6 for ApoL6-PIP2 still associates with LD by an alternate component of phospholipid monolayer and can inhibit lipolysis, suggesting that ApoL6-Plin1 interaction itself might play the major role in ApoL6 inhibition of lipolysis. In this regard, while there are 6 members of ApoL6 family, all containing common amphipathic helix, membrane domain, and leucine zipper domain, the overall sequence homology is not high. One family member is secreted and may be a component of high-density lipoproteins, while others are intracellular proteins that affect lipid movement or allow the binding of lipids to organelles^41,42^. ApoL6 has also been reported to be proapoptotic and ApoL6 was reported to interact with lipid/fatty acid components during apoptosis^43^.

### ApoL6 suppression of adipose lipolysis

Our in vitro and in vivo studies reveal the importance of ApoL6 function to inhibit lipolysis. Even through ApoL6 directly interacts with only Plin1 to interrupt HSL-Plin1 binding, we did not detect DAG accumulation in ApoL6 overexpressing adipocytes or WAT of ApoL6-TG mice, indicating ApoL6 inhibition of overall lipolysis (Fig. 1l). Plin1 is known to have dual function by augmenting stimulated lipolysis, while suppressing basal lipolysis: Thus, several studies have shown that Plin1 knockdown decreases lipolysis at stimulated condition, indicating the requirement of Plin1 for stimulated lipolysis^11^. Expression of ApoL6 in the fasted state is suppressed via transcriptional mechanism, and absence of ApoL6 allows interaction of phosphorylated HSL to Plin1 which is phosphorylated at the N-terminal region. Artificial overexpression of ApoL6 competes with HSL binding to Plin1 to inhibit lipolysis. Deletion of ApoL6 C-terminal domain or KD of Plin1 diminished ApoL6 inhibitory effect on lipolysis demonstrating the importance of ApoL6-Plin1 binding for ApoL6 function. Under the basal or fed condition, however, Plin1 plays a crucial role in restricting adipose lipolysis. In vivo and in vitro studies, including those on Plin1-null mice, have shown that reduced levels of Plin1 or absence of Plin1 results in increased basal lipolysis^35,36,44^. Plin1 binds CGI-58 with high affinity and thereby suppresses CGI-58-ATGL interaction^10^. The C-terminal domain of Plin1 has been shown to be essential for binding to CGI-58, and this interaction stabilizes CGI-58 localization on the LD^45^. Adipocytes overexpressing mutant Plin1 exhibited elevated basal lipolysis. Thus, expression of Plin1 frameshift mutations that lack C-terminal amino acid sequence or Plin1 with C-terminal truncation could reduce TAG storage in cells by increasing TAG turnover^46^. Here, we show that, in the fed condition, ApoL6 is transcriptionally activated by USF-1 and SREBP1c and increased ApoL6 levels inhibit lipolysis by blocking interaction of N-terminal domain of Plin1 to HSL. In the basal condition, artificial overexpression of ApoL6 did not increase basal lipolysis, since ApoL6 interacting domain of Plin1 resides at N-terminus, but not C-terminus where CGI-58 interacts. Future studies are needed to understand how ApoL6 could inhibit lipolysis in basal condition in cultured adipocytes. Physiologically, although ApoL6 decreases during fasting, overexpression of ApoL6 in transgenic mice had greater inhibitory effect on lipolysis in stimulated condition. We propose that ApoL6 protein levels decrease in fasted condition, potentially involving ApoL6 protein degradation mechanism, to allow Plin1-HSL interaction, resulting in stimulation of lipolysis. The first step of lipolysis in basal and stimulated states is catalyzed by ATGL. Triglyceride hydrolytic activity increases in stimulated state upon ATGL S406 phosphorylation and interaction with CGI58^13,47^. Although we did not detect direct interaction between ApoL6, the fact that we did not detect DAG accumulation in ApoL6 overexpressing cells and in WAT of ApoL6 TG mice (Fig. 1l) suggests that, not only HSL but ATGL function might also be altered by ApoL6. Immunoblotting using ATGL S406 antibody showed greatly lower ATGL phosphorylation in WAT of ApoL6-TG mice in fasted (stimulated) condition (Fig. 6f), suggesting a decrease of ATGL activity upon ApoL6 overexpression. In this regard, our Co-IP showed ApoL6 interaction, not only with Plin1 but also with HSL and ATGL. However, neither HSL nor ATGL directly interacts with ApoL6 implicating a large lipolytic complex formation. While we clearly demonstrate how ApoL6 interaction with Plin1 may affect HSL activity, the underlying molecular details of ApoL6 effect on lipolysis, including that on ATGL, need to be further studied in the future.

### Effect of ApoL6 on adiposity, insulin resistance and FLD

Our global ApoL6 KO mice showed protection from diet-induced obesity, while mice overexpressing ApoL6 in adipose tissue showed exacerbated adiposity. ApoL6 expression is increased in WAT of HFD-induced and genetic obesity mouse models (Previous reported downregulation of ApoL6 during adipocyte differentiation and genetic obesity were not reproducible in our hands^13,48^. Thus, ApoL6 knockout mice showed improved glucose tolerance and insulin sensitivity while ApoL6 overexpressing mice had impaired glucose tolerance and insulin resistance. These changes in insulin sensitivity reflect the inhibitory effect of ApoL6 on TAG accumulation in adipose tissue. In this regard, Plin1 KO mice^35,36,49^ had lower adipose mass and were resistant to diet-induced obesity but developed glucose intolerance and insulin resistance. These mice also had higher serum TAG but normal serum FFA levels^49^. Although the authors did not examine liver TAG accumulation, fatty liver disease (FLD) might have contributed to insulin resistance in these mice. Human Plin1 deficiency in heterozygous frameshift mutations had partial lipodystrophy with dyslipidemia and insulin resistance/diabetes^46^. Interestingly, however, Plin1 overexpression in adipose tissue has been reported also to protect mice against diet-induced obesity by inhibiting basal and stimulated lipolysis and to improve glucose tolerance^44^. In our study, probably due to changes in adiposity, decreasing lipolysis in adipocytes can bring insulin resistance, while increasing lipolysis can improve insulin sensitivity.

Higher lipolysis in WAT of KO mice raises a question of whether there could be ectopic fat storage. But we did not find any ectopic fat accumulation in liver or muscle. In fact, we not only detected less crown-like structures and lower expression of inflammatory markers in WAT of KO mice (Supplementary Fig. 3e left), but we also detected improved insulin response in liver and muscle from KO mice (Supplementary Fig. 3e, f). Moreover, in our ex vivo experiment using dispersed adipocytes from WAT of KO mice, we detected higher FA oxidation, indicating that increased FA generated from lipolysis, at least in part, might be oxidized within WAT (Supplementary Fig. 3h). In this regard, controversial results were reported with HSL-deficient mice: Some HSL-deficient mouse models were shown to develop FLD^50,51^, and adipose-specific deletion of HSL causing FLD by 8 months of age^52^. Other mouse models were reported to have a low hepatic TAG content^7,53,54^. However, loss of function of HSL by mutations in humans was reported to bring FLD^55–57^. Interestingly, while ApoL6 is present only in WAT in young mice, ApoL6 was detected also in the liver, mainly in hepatocytes, of mice upon HFD feeding and upon aging, although at a greatly lower level than WAT (Supplementary Fig. 4h, l). These observations suggest that increased TAG accumulation in the liver upon HFD feeding or aging may have induced ApoL6 expression, contributing to glucose intolerance and insulin resistance. The Human Protein Atlas (https://www.proteinatlas.org) indicates the presence of ApoL6, not only in WAT, but also in liver. However, we found ApoL6 primarily in adipose tissue of young mice on normal chow diet. This discrepancy might be due to diet and aging. Considering the GWAS data of ApoL6 SNP association with triglyceride levels in humans, ApoL6 may provide a future therapeutic target against obesity/diabetes and FLD.

## Methods

### Resources

#### Mouse models

ApoL6 transgenic mice: Two strains of ApoL6 overexpressing mice were generated, Myc-tagged ApoL6 driven by −5.4 kb aP2 (FABP4) promoter (aP2-ApoL6 TG) and HA-tagged ApoL6 driven by the −5.2 kb adiponectin promoter (adipoQ-ApoL6 TG). Mice were genotyped using their tails. Positive mice were mated with C57BL/6 J mice (The Jackson Laboratory) and both lines were germline transmitted. All transgenic mice and littermates were on C57BL/6 J background. aP2-ApoL6 TG genotype primers were forward 5′-AAACCCAACAGAGCTTGCAG-3′ and reverse 5′-CAAGTCCTCTTCAGAAATGAG-3′. adipoQ-ApoL6 TG genotype primers were forward 5′- TGTCAATTTCAGGGCTCAGGATA-3′ and 5′-GCTTTCGTCAATGTGGTCTGC-3′.

Global ApoL6 knockout mice (ApoL6 KO) were generated by using the CRISPR-Cas9 system, gRNA targeting translation start site (TCTGTCTGATGGCTCGGGGTTGG) and Cas9 mRNA were injected into the zygotes. By sequencing the ApoL6 genomic region of KO cells, we verified one KO line with a 11 bp deletion in the coding region of the third exon (18 bp after translation start site) and the line was germline transmitted (Fig. 2a upper). Transgenic and knockout mice were generated by the UC Berkeley Gene Targeting Facility. Primers for wild type were forward 5′- AAACTCCAACCCCGAGCCAT-3′ and reverse 5′- GCCCCAATTAAACGCTTTCC-3′; ApoL6 KO mutant primers were forward 5′- GGTGCAAACTCCATCAGA-3′ and reverse 5′- GCCCCAATTAAACGCTTTCC-3′.

We provided either a standard chow diet (Harlan Teklad LM-485) or a high-fat diet (HFD; 45% of calories from fat, 35% of calories from carbohydrates, and 20% of calories from protein; Research Diets) *ad libitum* after weaning. For fasting/feeding experiments, mice were fasted overnight and then fed a high carbohydrate, fat-free diet (70 kcal% carbohydrates, Research Diets) for 8–16 h. The light was turned on at 7 a.m. and off at 7 p.m. Animal housing and all protocols and experimental procedures were approved by the University of California at Berkeley Animal Care and Use Committee.

#### GTT and ITT

For GTTs, mice were fasted overnight, and glucose (2 mg/g) was administered intraperitoneally. For ITTs, mice were fasted for 4 h, and insulin (0.75 U/kg) was administered.

#### Analysis of insulin signaling

After an overnight fast, WT and KO mice were intraperitoneally injected with saline or 0.85 units/kg insulin and were sacrificed 10 min after. Lysates from WAT and liver were obtained in homogenization buffer (150 mmol/l NaCl, 1% Triton X-100, 0.5% sodium deoxycholate, 0.1% SDS, 10 mmol/l Tris, pH 7.4) containing a protease and phosphatase inhibitor cocktail (Sigma). Proteins (30–60 μg) were subjected to SDS-PAGE and immunoblotted with specific antibodies for total Akt or Akt phosphorylated (p-AKT) at residue Ser473 (Cell Signaling).

#### Cell culture and adipocyte differentiation

All cells were cultured at 37 °C in high-glucose Dulbecco’s modified Eagle’s medium (Gibco BRL) supplemented with 10% (v/v) heat-inactivated fetal bovine serum (Omega) and 100 U/ml penicillin/streptomycin (Gibco-BRL).

MEFs were prepared from E13.5 embryos. Briefly, embryos were removed and separated from maternal tissues and yolk sacs were finely minced, digested with 0.25% for 30 min at 37 °C, and centrifuged for 5 min at 1000 × *g*. The pellet was resuspended in culture medium before plating. All differentiation experiments were carried out using cells at passage 2. For adipocyte differentiation of MEFs, MEFs were split into six-well plates and cultured to confluence. Two days later, the medium was replaced with differentiation induction medium containing 0.5 mM methyl-isobutyl-xanthine (MIX), 1 μM dexamethasone (DEX), 10 μg/ml insulin, 10 μM troglitazone, and 10% (v/v) FBS. MEFs were treated with differentiation agents for 4 days, and the medium was renewed every other day.

For 3T3-L1 adipocyte differentiation, 3T3-L1 cells (ATCC, CL-173) were split into six-well plates and cultured to confluence. Two days later, the medium was replaced with differentiation induction medium containing 0.5 mM MIX, 1 μM DEX, 10 μg/ml insulin and 10% (v/v) FBS for 2 days and changed back to complete media for additional two days.

Human fibroblast cell line was purchase from ATCC (ATCC R PCS-210-010). There was no gender information available.

#### Adenovirus and lentivirus generation and transduction and plasmid transfection

Adenoviral constructs were generated using ApoL6 full length plasmid by Vector Biolabs. Lentiviral constructs were generated by subcloning ApoL6 and its mutant into pLenti6/V5-D-TOPO vector and inducible expressing vector, pInducer 20 (Addgene). ApoL6 was also subcloned into pcDNA3.1. Lentiviral Plin1 shRNA plasmid was purchased from Sigma. Lentivirus was first packaged in 293 FT cells using co-transfection with LV-MAX Lentiviral packaging mix (ThermoFisher) for 48 h, and cells were infected with harvested lentivirus for 48 h before further experiments. The 293FT cells were transfected with-444-FAS-Luc along with various expression plasmids, including USF-1 and SREBP1c, by using Lipofectamine 2000 (Invitrogen). Luciferase assays were performed using Dual-Luc reagent (Promega).

#### Mass spec analysis

WATs from adipoQ TG mice (overexpressing ApoL6) were minced and lysates were cross-linked using NHS-Diazirine (SDA, ThermoFisher #26167). LD-associated proteins were isolated after crosslinking. Total cell lysates from differentiated 3T3-L1 cells overexpressing ApoL6-HA were crosslinked. Tissues and cells in lysis buffer (10 mM HEPES, 1 mM EDTA, PH 7.4 and 1% *n*-dodecyl-β-D-maltoside (DDM) with proteinase cocktail) were homogenized by sonication and centrifuged at 20,000 g for 1 h on ice. Supernatants were subjected to IP with HA antibody using HA magnetic beads. After washing with PBST (Phosphate buffered saline (pH 7.4) or Tris buffered saline, pH 7.4, containing 0.05% Tween-20, for 3 times, proteins were eluted with 1 M Glycine pH 2.5, followed by addition of 10% neutralization buffer (1 M Tris Buffer, PH 9.0). The eluates were diluted with lysis buffer and IP with either HSL antibody or Plin1 antibody and subjected to A/G magnetic beads. After washing 3 times with PBST, proteins were eluted with native PAGE sample buffer containing 5% digitonin and 1% DDM (Invitrogen BN2003) and subjected to nondenaturing PAGE. The gels were subjected to immunoblotting, or the bands were excised out for MS analysis. MS analysis was performed by the Vincent J. Coates Proteomics/Mass Spectrometry Laboratory (P/MSL) at UC Berkeley.

#### Bimolecular fluorescence complementation (BiFC) assay

We employed BiFC based on fluorescence detection upon complementation between nonfluorescent N- and C-fragment fused to two different proteins that upon interaction of the two proteins becomes fluorescent. We used Venus system (Addgene) by fusing VN210 (Venus aa1-210) with Plin1 (Plin1-nVenus) and VC210 (Venus aa210-238) with ApoL6 (ApoL6-cVenus) and the two constructs were transfected into differentiating 3T3-L1 adipocytes, and the interactions were visualized by fluorescence imaging.

#### Northern blot analysis, RT-PCR and RT-qPCR

Total RNA prepared with Trizol (Invitrogen) were subjected to agarose-formaldehyde gel electrophoresis and blotted onto Hybond N membranes (Amersham). The membranes were hybridized with ^32^P-labeled cDNAs for mouse ApoL6 or FAS. Primer sets used for RT-PCR to produce the cDNA probes were described in supplemental data section. Hybridization was performed as described previously (Wang and Sul, 2006). For RT-qPCR, 1 μg of total RNA was reverse transcribed using SuperScript II (Invitrogen). The cDNAs were mixed with SYBR^TM^ green PCR master mix (Invitrogen) and specific primers for ApoL6, Pref-1, C/EBPα or PPARγ. Data were analyzed by using ABI7900. Gene expression levels were calculated by normalization to glyceraldehyde 3-phosphate dehydrogenase (GAPDH) by the ΔΔ*C*_*T*_ method. The mean cycle threshold (*C*_*T*_) was converted to a relative expression value by the 2^−ΔΔ*CT*^ method, and the range was calculated by the 2^−(ΔΔ*CT* + standard deviation of ΔΔ*CT*)^ method.

#### Immunoblotting

Total lysates were subjected to 8% or 10% SDS-PAGE, transferred to Protran membranes. After blocking with 4% nonfat dry milk in Tris-buffered saline-Tween buffer, the membranes were probed with first antibodies and followed by a horseradish peroxidase (HRP)-conjugated secondary antibody (Bio-Rad). Blots were visualized by enhanced chemiluminescence (PerkinElmer), and images were captured with Bio-Rad ChemiDoc imaging system.

#### Isolation of LD-associated proteins

LD-associated proteins were isolated by using single centrifugation method^58^. Briefly, 50–100 mg epididymal fat from mice with 800 ul lysis buffer (10 mM HEPES and 1 mM EDTA, PH 7.4) was minced with a razor blade and homogenized. 200 ug of 60% (w/w) sucrose was added to the tissue sample and incubated on ice for 20 min and mixed well. 600 ul lysis buffer with food dye (2ul/1 ml of lysis buffer) was carefully layered on top of the homogenate and centrifuged for 2 h at 20,000 g at 4 °C. The tube was frozen thoroughly, and associated proteins were harvested from top layer with RIPA buffer containing phosphatase inhibitor cocktail (Cell Signaling) and protease inhibitor cocktail (Sigma).

#### Immunoprecipitation and GST pull-down

Total LD-associated proteins from differentiated 3T3-L1 cells in a buffer containing 1% Triton X-100, 150 mM NaCl, 10% glycerol, 25 mM Tris [pH 7.4], phosphatase inhibitor cocktail (Cell Signaling), and protease inhibitor cocktail (Sigma) were incubated with antibodies against Plin1 at 4 °C overnight, followed by the addition of 40 μl of protein A/G-agarose beads (Santa Cruz) and incubation for 1 h. Beads were collected by pulse centrifugation, washed five times with lysis buffer, and heated to 99 °C for 5 min in 5× SDS sample buffer. The input and the immunoprecipitated fraction were subjected to SDS-PAGE. HSL, Plin1, and GAPDH were detected by immunoblotting with each antibody.

For GST pull-down, bacterially expressed GST-ApoL6 fusion protein on glutathione-agarose beads (Santa Cruz) was incubated with ^35^S labeled proteins made by using TNT Quick Coupled transcription/Translation System (Promega) with ^35^S labeled methionine. The proteins were separated by SDS-PAGE before autoradiography.

#### Lipolysis assay in cultured adipocytes and in mice

12 well plates of differentiated 3T3-L1 adipocytes were washed with Krebs Ringer buffer (KRB; 12 mM HEPES, 121 mM NaCl, 4.9 mM KCl, 1.2 mM MgSO_4_, 0.33 mM CaCl_2_) and incubated at 37 °C in 300 μl of Krebs Ringer buffer containing 2% FA-free bovine serum albumin (BSA) and 0.1% glucose in the presence or absence of 10 μM isoproterenol (Sigma) for 1 hr.

Primary adipocytes were isolated from epi-WAT after digestion at 37 °C for 1 h with collagenase (Roche) in KRB supplemented with 3 mM glucose and 1% FA-free BSA. Digestion products were filtered through nylon mesh and centrifuged. Adipocytes were collected from the upper phase. Cells were then incubated at 37 °C in 500 μl of KRB in the presence or absence of 10 μM isoproterenol (Sigma) for 1 h or with 50 μM ATGL inhibitor, Atglistatin (Sigma), and 10 μM HSL inhibitor CAY10499 (Cayman). Lipolysis was assayed by the release of FFAs and glycerol into the media as described previously using the NEFA-HR (Wako, #997-76491) and glycerol reagent (Sigma), respectively.

For in vivo lipolysis, mice were injected intraperitoneally with isoproterenol in PBS at 10 mg/kg. Blood samples from tails were collected at 0, 15, 30, 60 and 90 min after injection. Serum was subjected to glycerol measurement.

#### Extraction of Lipids and measurement of TAG and DAG

3T3-L1 adipocytes were pulse-labeled with [U-^14^C] palmitic acid at a final concentration of 0.15 μCi/ml for 24 h. Cells were then switched to label-free media and infected with control or ApoL6 adenovirus. Lipids were extracted from cells by the method of Bligh and Dyer and separated by TLC using hexane/diethyl ether/acetic acid (80:20:2), after which radioactive lipids were detected by autoradiography. ^14^C-TAG was quantified by scraping of the corresponding band into CytoScint (Fisher Scientific) followed by scintillation counting. Also, Total neutral lipids were extracted from cells or liver and solubilized in 1% tritonX-100. TAG were measured using TAG assay kit (Abcam, #ab65336). DAG were measured using DAG assay kit (Cell biolabs, Inc MET-5028).

#### Serum metabolite measurements

Blood was centrifuged at 4 °C for 15 min for fractionation. TAG levels were measured with the Infinity triglyceride reagent (Thermo-Fisher), and non-esterified FAs (NEFAs) were measured with the NEFA-HR kit (Wako). TAG contents were measured with Triglyceride quantification assay kit (Abcam, #ab65336). Total cholesterol and HDL and LDL cholesterol were measured with Cholesterol assay kit (Abcam ab65390).

#### Protein-lipid overlay assay

Membrane lipid strips ((p-6002) Echelon biosciences) were blocked in buffer containing PBS, 0.1% Tween 20, 3% BSA for 1 h. The strips were then probed with ApoL6-GST for 4 h. The strips were washed 3 times with a buffer containing PBS and 0.1% Tween20 and then probed with GST antibody followed by anti-rabbit HRP conjugate as the secondary antibody. The strips were developed with Pierce ECL detection kit (Thermo-Fisher).

#### Adipocyte size determination

Epi-WAT samples were fixed in 10% buffered formalin, embedded in paraffin, cut into 8-μm-thick sections, and stained with hematoxylin and eosin. Adipocyte size was determined with NIH ImageJ software by measuring a minimum of 300 cells per slide, five slides per sample, and three or four mice per group.

### Statistics and reproducibility

The results were expressed as means ± the standard deviation (SD). Student’s *t* test was used for comparisons of two groups, multiple *t* test was used for multiple groups. Multiple *t* test and two-way ANOVA were used for lipolysis. Two-way ANOVA was used for GTT and ITT and body weight. All significance levels were set at *P* < 0.05. All experiments were repeated at least twice, and representative data were shown.

### Reporting summary

Further information on research design is available in the Nature Portfolio Reporting Summary linked to this article.

### Supplementary information

Supplementary Information

Reporting Summary

### Source data

Source Data

## Data Availability

Sources data are provided with this paper. Proteomics data for this paper are provided with the accession number PXD038334. Source data are provided with this paper.

## References

[1] Unger RH, Scherer PE (2010). Gluttony, sloth and the metabolic syndrome: a roadmap to lipotoxicity. Trends Endocrinol. Metab..

[2] Ahmadian M (2009). Adipose overexpression of desnutrin promotes fatty acid use and attenuates diet-induced obesity. Diabetes.

[3] Duncan RE (2010). Characterization of desnutrin functional domains: critical residues for triacylglycerol hydrolysis in cultured cells. J. Lipid Res..

[4] Jenkins CM (2004). Identification, cloning, expression, and purification of three novel human calcium-independent phospholipase A2 family members possessing triacylglycerol lipase and acylglycerol transacylase activities. J. Biol. Chem..

[5] Villena JA, Roy S, Sarkadi-Nagy E, Kim KH, Sul HS (2004). Desnutrin, an adipocyte gene encoding a novel patatin domain-containing protein, is induced by fasting and glucocorticoids: ectopic expression of desnutrin increases triglyceride hydrolysis. J. Biol. Chem..

[6] Zimmermann R (2004). Fat mobilization in adipose tissue is promoted by adipose triglyceride lipase. Science.

[7] Haemmerle G (2002). Hormone-sensitive lipase deficiency in mice causes diglyceride accumulation in adipose tissue, muscle, and testis. J. Biol. Chem..

[8] Taschler U (2011). Monoglyceride lipase deficiency in mice impairs lipolysis and attenuates diet-induced insulin resistance. J. Biol. Chem..

[9] Granneman JG (2007). Analysis of lipolytic protein trafficking and interactions in adipocytes. J. Biol. Chem..

[10] Granneman JG, Moore HP, Krishnamoorthy R, Rathod M (2009). Perilipin controls lipolysis by regulating the interactions of AB-hydrolase containing 5 (Abhd5) and adipose triglyceride lipase (Atgl). J. Biol. Chem..

[11] Sztalryd C (2003). Perilipin A is essential for the translocation of hormone-sensitive lipase during lipolytic activation. J. Cell Biol..

[12] Yamaguchi T, Omatsu N, Matsushita S, Osumi T (2004). CGI-58 interacts with perilipin and is localized to lipid droplets. Possible involvement of CGI-58 mislocalization in Chanarin-Dorfman syndrome. J. Biol. Chem..

[13] Ahmadian M (2011). Desnutrin/ATGL is regulated by AMPK and is required for a brown adipose phenotype. Cell Metab..

[14] Kim SJ (2016). AMPK phosphorylates Desnutrin/ATGL and hormone-sensitive lipase to regulate lipolysis and fatty acid oxidation within adipose tissue. Mol. Cell Biol..

[15] Yin W, Mu J, Birnbaum MJ (2003). Role of AMP-activated protein kinase in cyclic AMP-dependent lipolysis In 3T3-L1 adipocytes. J. Biol. Chem..

[16] Garcia A, Sekowski A, Subramanian V, Brasaemle DL (2003). The central domain is required to target and anchor perilipin A to lipid droplets. J. Biol. Chem..

[17] Sahu-Osen A (2015). CGI-58/ABHD5 is phosphorylated on Ser239 by protein kinase A: control of subcellular localization. J. Lipid Res.

[18] Miyoshi H (2007). Control of adipose triglyceride lipase action by serine 517 of perilipin A globally regulates protein kinase A-stimulated lipolysis in adipocytes. J. Biol. Chem..

[19] Miyoshi H (2006). Perilipin promotes hormone-sensitive lipase-mediated adipocyte lipolysis via phosphorylation-dependent and -independent mechanisms. J. Biol. Chem..

[20] Shin H (2017). Lipolysis in brown adipocytes is not essential for cold-induced thermogenesis in mice. Cell Metab..

[21] Clifford GM, Londos C, Kraemer FB, Vernon RG, Yeaman SJ (2000). Translocation of hormone-sensitive lipase and perilipin upon lipolytic stimulation of rat adipocytes. J. Biol. Chem..

[22] Egan JJ (1992). Mechanism of hormone-stimulated lipolysis in adipocytes: translocation of hormone-sensitive lipase to the lipid storage droplet. Proc. Natl Acad. Sci. USA.

[23] Osuga J (2000). Targeted disruption of hormone-sensitive lipase results in male sterility and adipocyte hypertrophy, but not in obesity. Proc. Natl Acad. Sci. USA.

[24] Su CL (2003). Mutational analysis of the hormone-sensitive lipase translocation reaction in adipocytes. J. Biol. Chem..

[25] Karlsson M, Contreras JA, Hellman U, Tornqvist H, Holm C (1997). cDNA cloning, tissue distribution, and identification of the catalytic triad of monoglyceride lipase. Evolutionary relationship to esterases, lysophospholipases, and haloperoxidases. J. Biol. Chem..

[26] Begum N (1995). Stimulation of protein phosphatase-1 activity by insulin in rat adipocytes. Evaluation of the role of mitogen-activated protein kinase pathway. J. Biol. Chem..

[27] Brady MJ, Saltiel AR (2001). The role of protein phosphatase-1 in insulin action. Recent Prog. Horm. Res.

[28] Clifford GM, McCormick DK, Londos C, Vernon RG, Yeaman SJ (1998). Dephosphorylation of perilipin by protein phosphatases present in rat adipocytes. FEBS Lett..

[29] Schweiger M, Lass A, Zimmermann R, Eichmann TO, Zechner R (2009). Neutral lipid storage disease: genetic disorders caused by mutations in adipose triglyceride lipase/PNPLA2 or CGI-58/ABHD5. Am. J. Physiol. Endocrinol. Metab..

[30] Schweiger M (2017). Pharmacological inhibition of adipose triglyceride lipase corrects high-fat diet-induced insulin resistance and hepatosteatosis in mice. Nat. Commun..

[31] Yang A, Mottillo EP (2020). Adipocyte lipolysis: from molecular mechanisms of regulation to disease and therapeutics. Biochem J..

[32] Yang X (2010). The G(0)/G(1) switch gene 2 regulates adipose lipolysis through association with adipose triglyceride lipase. Cell Metab..

[33] Qiao L, Kinney B, Schaack J, Shao J (2011). Adiponectin inhibits lipolysis in mouse adipocytes. Diabetes.

[34] Zhang HH (2003). Lipase-selective functional domains of perilipin A differentially regulate constitutive and protein kinase A-stimulated lipolysis. J. Biol. Chem..

[35] Martinez-Botas J (2000). Absence of perilipin results in leanness and reverses obesity in Lepr(db/db) mice. Nat. Genet.

[36] Tansey JT (2001). Perilipin ablation results in a lean mouse with aberrant adipocyte lipolysis, enhanced leptin production, and resistance to diet-induced obesity. Proc. Natl Acad. Sci. USA.

[37] Truebestein L, Leonard TA (2016). Coiled-coils: the long and short of it. Bioessays.

[38] Greenberg AS (1993). Isolation of cDNAs for perilipins A and B: sequence and expression of lipid droplet-associated proteins of adipocytes. Proc. Natl Acad. Sci. USA.

[39] Sztalryd C, Brasaemle DL (2017). The perilipin family of lipid droplet proteins: gatekeepers of intracellular lipolysis. Biochim Biophys. Acta Mol. Cell Biol. Lipids.

[40] Souza SC (2002). Modulation of hormone-sensitive lipase and protein kinase A-mediated lipolysis by perilipin A in an adenoviral reconstituted system. J. Biol. Chem..

[41] Duchateau PN (2000). Plasma apolipoprotein L concentrations correlate with plasma triglycerides and cholesterol levels in normolipidemic, hyperlipidemic, and diabetic subjects. J. Lipid Res.

[42] Pant J (2021). Apolipoproteins L1-6 share key cation channel-regulating residues but have different membrane insertion and ion conductance properties. J. Biol. Chem..

[43] Liu Z, Lu H, Jiang Z, Pastuszyn A, Hu CA (2005). Apolipoprotein l6, a novel proapoptotic Bcl-2 homology 3-only protein, induces mitochondria-mediated apoptosis in cancer cells. Mol. Cancer Res.

[44] Miyoshi H (2010). Perilipin overexpression in mice protects against diet-induced obesity. J. Lipid Res.

[45] Gandotra S (2011). Human frame shift mutations affecting the carboxyl terminus of perilipin increase lipolysis by failing to sequester the adipose triglyceride lipase (ATGL) coactivator AB-hydrolase-containing 5 (ABHD5). J. Biol. Chem..

[46] Gandotra S (2011). Perilipin deficiency and autosomal dominant partial lipodystrophy. N. Engl. J. Med..

[47] Pagnon J (2012). Identification and functional characterization of protein kinase A phosphorylation sites in the major lipolytic protein, adipose triglyceride lipase. Endocrinology.

[48] Tan Y (2019). miR-10b-5p regulates 3T3-L1 cells differentiation by targeting Apol6. Gene.

[49] Sohn JH (2018). Perilipin 1 (Plin1) deficiency promotes inflammatory responses in lean adipose tissue through lipid dysregulation. J. Biol. Chem..

[50] Mulder H (2003). Hormone-sensitive lipase null mice exhibit signs of impaired insulin sensitivity whereas insulin secretion is intact. J. Biol. Chem..

[51] Roduit R (2001). A role for hormone-sensitive lipase in glucose-stimulated insulin secretion: a study in hormone-sensitive lipase-deficient mice. Diabetes.

[52] Xia B (2017). Adipose tissue deficiency of hormone-sensitive lipase causes fatty liver in mice. PLoS Genet.

[53] Donnelly KL (2005). Sources of fatty acids stored in liver and secreted via lipoproteins in patients with nonalcoholic fatty liver disease. J. Clin. Invest.

[54] Voshol PJ (2003). Increased hepatic insulin sensitivity together with decreased hepatic triglyceride stores in hormone-sensitive lipase-deficient mice. Endocrinology.

[55] Albert JS (2014). Null mutation in hormone-sensitive lipase gene and risk of type 2 diabetes. N. Engl. J. Med..

[56] Fischer J (2007). The gene encoding adipose triglyceride lipase (PNPLA2) is mutated in neutral lipid storage disease with myopathy. Nat. Genet.

[57] Haemmerle G (2006). Defective lipolysis and altered energy metabolism in mice lacking adipose triglyceride lipase. Science.

[58] Harris LLS, Shew TM, Skinner JR, Wolins NE (2012). A single centrifugation method for isolating fat droplets from cells and tissues. J. Lipid Res..

