# Supplementary material for: ApoL6 associates with lipid droplets and disrupts Perilipin1-HSL interaction to inhibit lipolysis
Source: Nat Commun. 2024 Jan 2;15:186. doi: 10.1038/s41467-023-44559-3 (PMC10762002; doi:10.1038/s41467-023-44559-3)
Supplement: Supplementary file 1 — Supplementary Information [file 41467_2023_44559_MOESM1_ESM.pdf]

SUPPLEMENTAL

Mass spectrometry: Mass spectrometry was performed at the Proteomics/ Mass Spectrometry Laboratory at University of California, Berkeley. A nano LC column was packed in a 100-μm inner diameter glass capillary with an integrated pulled emitter tip. The column consisted of 10 cm of Polaris c18 5-μm packing material (Varian). The column was loaded and conditioned using a pressure bomb. The column was then coupled to an electrospray ionization source mounted on a Thermo-Fisher LTQ XL linear ion trap mass spectrometer. An Agilent 1200 HPLC equipped with a split line to deliver a flow rate of 1 μl/min was used for chromatography. Peptides were eluted with a 90-minute gradient from 100% buffer A to 60% buffer B. Buffer A was 5% acetonitrile/0.02% heptafluorobutyric acid (HBFA); buffer B was 80% acetonitrile/0.02% HBFA. Collision-induced dissociation and electron transfer dissociation spectra were collected for each *m/z*. Protein identification and quantification and analysis were done with Integrated Proteomics Pipeline-IP2 (Bruker Scientific LLC, Billerica, MA, http://www.bruker.com)) using ProLuCID/Sequest (1,2), DTASelect2 (3,4), and Census (5,6). Spectrum raw files were extracted into ms1 and ms2 files from raw files using RawExtract 1.9.9 (http://fields.scripps.edu/downloads.php) 10, and the tandem mass spectra were searched against mouse database (7, 8).

1) Xu, T. a fast and sensitive tandem mass spectra-based protein identification program. Mol Cell Proteomics. Molecular & Cellular Proteomics 5(10): S174–S174 (2006).

2) Xu T, Park SK, Venable JD, Wohlschlegel JA, Diedrich JK, Cociorva D, Lu B, Liao L, Hewel J, Han X, Wong CCL, Fonslow B, Delahunty C, Gao Y, Shah H, ProLuCID: An improved SEQUEST-like algorithm with enhanced sensitivity and specificity, J Proteomics. 2015

3) Cociorva, D., D, L. T. & Yates, J. R. Validation of tandem mass spectrometry database search results using DTASelect. Current protocols in bioinformatics / editorial board, Andreas D. Baxevanis … [et al.] Chapter 13, Unit 13 14, doi:10.1002/0471250953.bi1304s16 (2007).

4) Tabb, D. L., McDonald, W. H. & Yates, J. R., III. DTASelect and Contrast: Tools for Assembling and Comparing Protein Identifications from Shotgun Proteomics. Journal of Proteome Research 1, 21-26 (2002).

5) Park, S. K., Venable, J. D., Xu, T. & Yates, J. R., 3rd. A quantitative analysis software tool for mass spectrometry-based proteomics. Nat Methods 5, 319-322, doi:10.1038/nmeth.1195 (2008).

6) Park, S. K. et al. Census 2: isobaric labeling data analysis. Bioinformatics 30, 2208-2209, doi:10.1093/bioinformatics/btu151 (2014).

7). Eng JK, MacCormak AL, Yates JR 3rd. An approach to correlate tandem mass spectral data of peptides with amino acid sequences in a protein database. *J Am Soc Mass Spectrom*. 1994;5(11):976–989.

8). Tabb DL, McDonald WH, Yates JR 3rd. DTASelect and Contrast: tools for assembling and comparing protein identifications from shotgun proteomics. *J Proteome Res*. 2002;1(1):21–26

SFigure 1

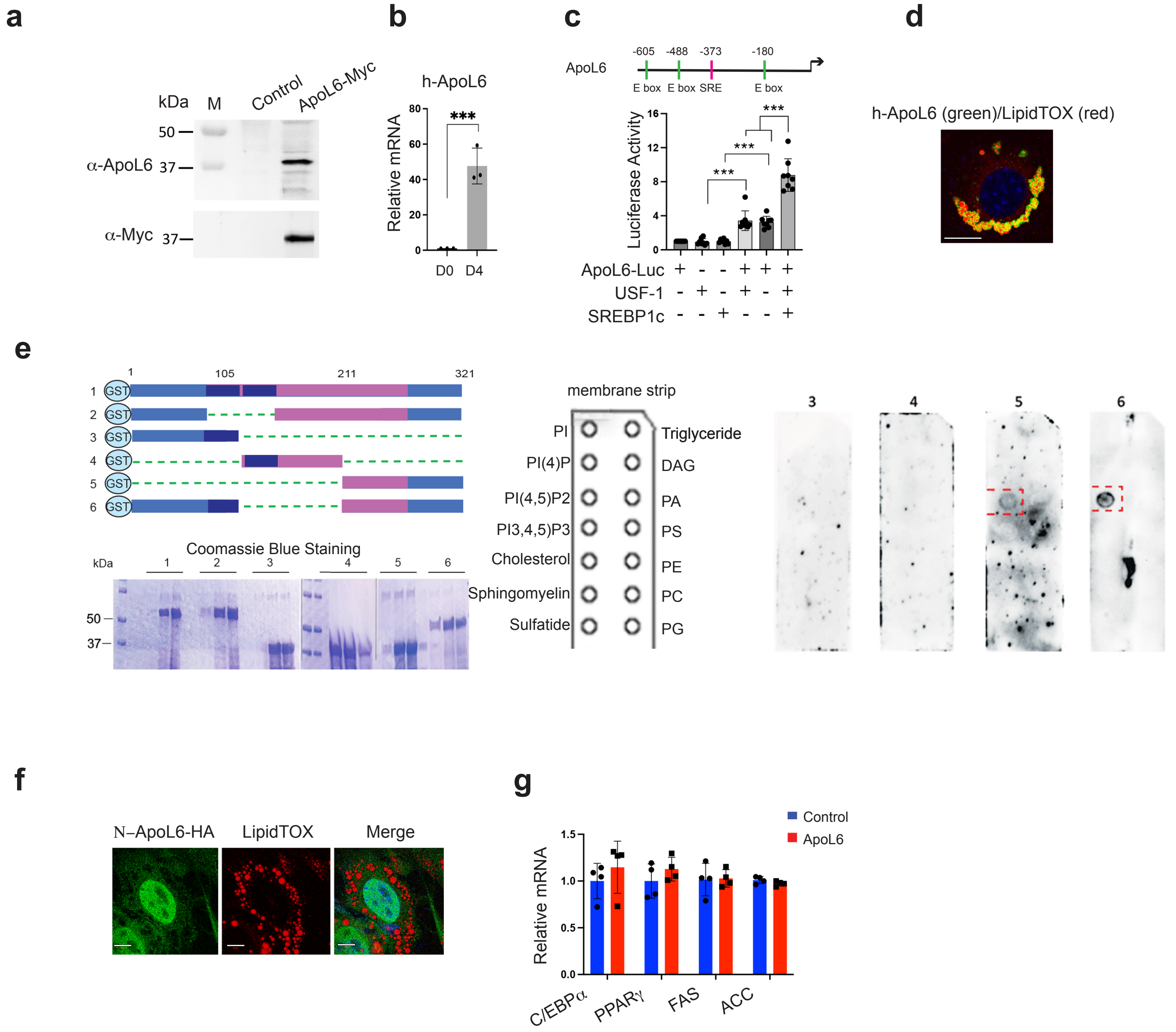

a
kDa
M
Control
ApoL6-Myc
50
37
α-ApoL6
α-Myc
b
h-ApoL6
Relative mRNA
***
D0
D4
c
ApoL6
-605
-488
-373
-180
E box
E box
SRE
E box
Luciferase Activity
***
ApoL6-Luc
USF-1
SREBP1c
d
h-ApoL6 (green)/LipidTOX (red)
e
1
105
211
321
GST
Coomassie Blue Staining
kDa
membrane strip
PI
PI(4)P
PI(4,5)P2
PI3,4,5)P3
Cholesterol
Sphingomyelin
Sulfatide
Triglyceride
DAG
PA
PS
PE
PC
PG
f
N–ApoL6-HA
LipidTOX
Merge
g
Control
ApoL6
Relative mRNA
C/EBPα
PPARγ
FAS
ACC

Supplementary Fig. 1. ApoL6 is expressed in adipocytes and localized on LD.
(**a**) Verification of custom-made ApoL6 antibody (peptide sequence: KDLKAANPTELA) by immunoblotting using lysates of HEK 292 cells transfected with ApoL6-Myc construct. (**b**) Human fibroblasts were differentiated into adipocytes. RT-qPCR for human ApoL6 mRNA levels before and after differentiation (Day 4, *n*=3, ***p<0.001, two tailed Student *t* test). Data represent mean ± SD. Independent experiments were repeated twice). (**c**) Location of E box and SRE motifs at ApoL6 promoter region (upper) and relative luciferase activity upon co-transfection of ApoL6 promoter-luciferase with USF-1 and SREBP-1c (lower, *n*=8, ***, p<0.001 compared to cells transfected with empty control vector, multiple *t* test). Data represent mean ± SD. Experiments were repeated twice. (**d**) Immunofluorescence using hApoL6 antibody followed by Alexa fluor 488 secondary antibody and merged with LipidTOX (red) staining in human adipocytes infected with hApoL6 lentivirus (bar=10μm). (**e**) Coomassie blue staining of purified GST-tagged full-length ApoL6 and its deletion constructs (left). Immunoblotting with GST antibody after incubation of lipid membrane strips with different deletions of ApoL6-GST (right). (**f**) 3T3-L1 adipocytes were infected with N-ApoL6-HA lentivirus. Immunofluorescence using HA antibody followed by Alexa fluor 488 secondary antibody (green) merged with LipidTOX (red) staining (bar=10μm). (**g**) RT-qPCR for expression of adipogenic transcription factors and lipogenic enzymes in differentiated 3T3-L1 adipocytes after infection with control or ApoL6 adenovirus (*n*=4, two tailed Student *t* test). Data represent mean ± SD. Experiments were repeated twice.

SFigure 2

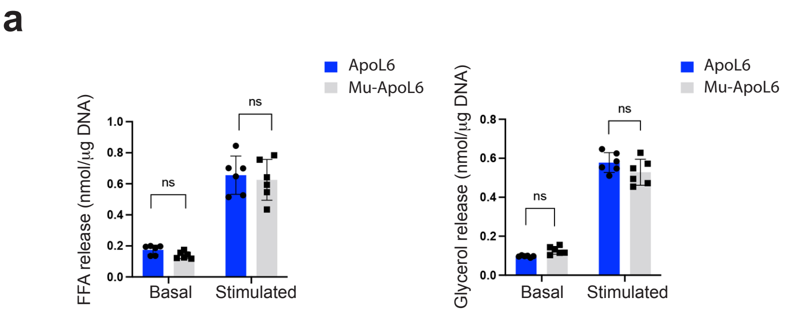
a
ApoL6
Mu-ApoL6
FFA release (nmol/μg DNA)
1.0
0.8
0.6
0.4
0.2
0.0
ns
ns
Basal
Stimulated
ApoL6
Mu-ApoL6
Glycerol release (nmol/μg DNA)
0.8
0.6
0.4
0.2
0.0
ns
ns
Basal
Stimulated

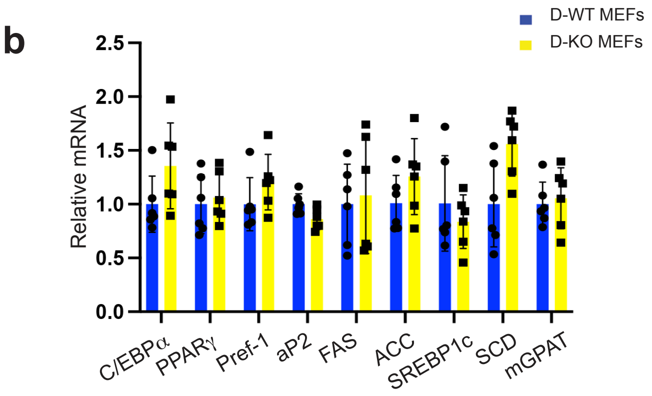
b
D-WT MEFs
D-KO MEFs
Relative mRNA
2.5
2.0
1.5
1.0
0.5
0.0
C/EBPα
PPARγ
Pref-1
aP2
FAS
ACC
SREBP1c
SCD
mGPAT

Supplementary Fig. 2. ApoL6 did not affect adipocyte differentiation.
(**a**) FFA and glycerol release from media of differentiated 3T3-L1 adipocytes infected either with lentiviral ApoL6 or lentiviral Mu-ApoL6 in basal and isoproterenol treated conditions (n=6, multiple t test). Data represent mean ± SD. Experiments were repeated twice. (**b**) RT-qPCR for expression of adipogenic transcription factors and lipogenic enzymes after differentiation of MEF into adipocytes (n=6). Data represent mean ± SD.

SFigure 3

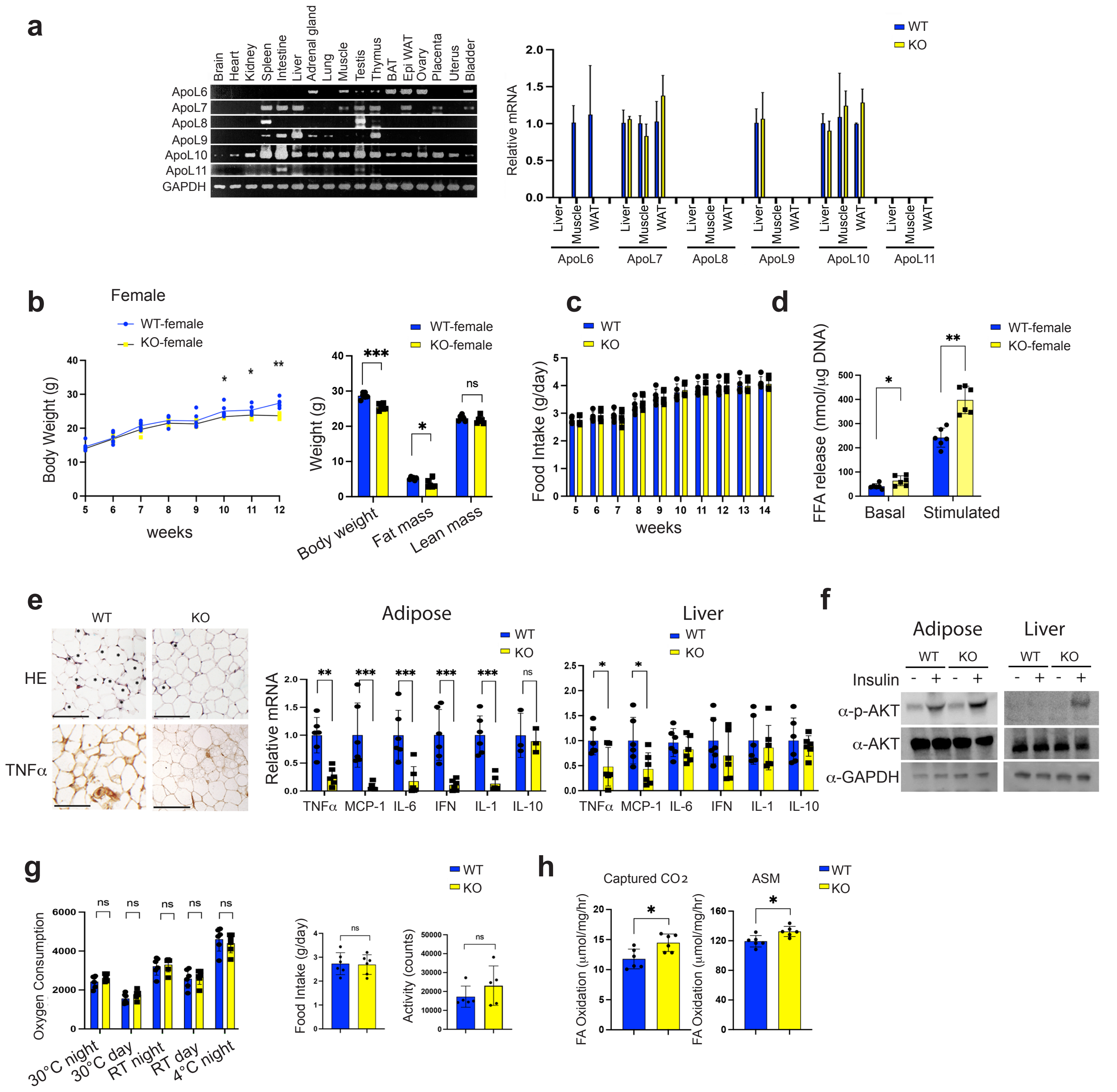

a
Brain Heart Kidney Spleen Intestine Liver Adrenal gland Lung Muscle Testis Thymus BAT Epi WAT Ovary Placenta Uterus Bladder
ApoL6
ApoL7
ApoL8
ApoL9
ApoL10
ApoL11
GAPDH
WT
KO
Relative mRNA
Liver Muscle WAT
ApoL6 ApoL7 ApoL8 ApoL9 ApoL10 ApoL11
b
Female
WT-female
KO-female
Body Weight (g)
weeks
Weight (g)
Body weight
Fat mass
Lean mass
ns
c
WT
KO
Food Intake (g/day)
weeks
d
WT-female
KO-female
FFA release (nmol/µg DNA)
Basal
Stimulated
e
WT
KO
HE
TNFα
Adipose
Liver
Relative mRNA
TNFα MCP-1 IL-6 IFN IL-1 IL-10
f
Adipose
Liver
Insulin
α-p-AKT
α-AKT
α-GAPDH
g
Oxygen Consumption
30°C night
30°C day
RT night
RT day
4°C night
Food Intake (g/day)
Activity (counts)
h
Captured CO2
ASM
FA Oxidation (µmol/mg/hr)

Supplementary Fig. 3. ApoL6 ablation in mice led to lower WAT mass and less inflammation in WAT and liver after HFD

(**a**) RT-PCR for ApoL6-11 expression in various tissues in mice (left). ApoL6 was expressed mainly in WAT. ApoL7 and 10 also expressed in WAT. ApoL7, 9 and 10 were detected in liver, and ApoL7 and 10 in muscle. RT-qPCR for ApoL6-11 expression in the liver, muscle, and WAT, showed no differences between WT and KO, indicating no compensation from other ApoL family members in KO. Data were presented as fold-change compared to WT (n=3) (right). (**b**) BW of female mice (n=6, *p=0.038, 0.012, ***p<0.001, Two-way AVNOVA test) during HFD feeding and EchoMRI (n=6, *p=0.02. ***p<0.001, multiple t test). (**c**) Food intake during HFD feeding (n=6). (**d**) FFA release from dispersed adipocytes isolated from female WAT (n=6, *p=0.012, ** p=0.0017). Data represent mean ± SD. (**e**) H&E staining and immunostaining for TNFα (left) of WAT. H&E staining showed less crown-like structures (labeled *) in KO than WT. TNFα immunostaining (brown) showed less in KO than WT (bar=100 μm). RT-qPCR for inflammatory markers after HFD (right) showed less expression of inflammation in WAT (n=6, **p=0.0016, ***p<0.001) and liver (n=6, *p=0.023 and 0.033) in KO. (**f**) Immunoblotting of WAT and liver samples after 10 min of insulin treatment (right), showing improved insulin response in WAT and liver of KO compared to WT. (**g**) Oxygen consumption rates (OCR) were measured using the Comprehensive Laboratory Animal Monitoring System (CLAMS). Mice were individually caged and maintained under a 12 hr light/12 hr dark cycle at different temperatures. Data were normalized to lean body mass determined by EchoMRI (n=5 mice per group). Food consumption and locomotor activity were shown. CLAMS study showed there was no significant differences in OCR between WT and KO mice at ambient temperature, 30°C or 4°C, suggesting no changes in thermogenic function by ApoL6 ablation. There were no significant changes in activities (n=5). Data represent mean ± SD. (**h**) FA oxidation in dispersed adipocytes from WAT. Cells were incubated with [U-^{14}C] palmitic acid (0.2 µCi/ml) for 1 h at 37ºC with gentle shaking. The buffer was acidified with 0.25 ml of H_2SO_4 (5N) and maintained sealed at 37ºC for an additional 60 min. Trapped radioactivity as $^{14}CO_2$ (complete palmitate oxidation) and ^{14}C-ASM (acid soluble metabolites; incomplete palmitate oxidation) was determined via liquid scintillation counting using Uniscint BD (National Diagnostics, Atlanta, GA). FA oxidation showed more in WAT of KO than WT (n=6, *p=0.0133 and 0.0104). Data represent mean ± SD.

SFigure 4

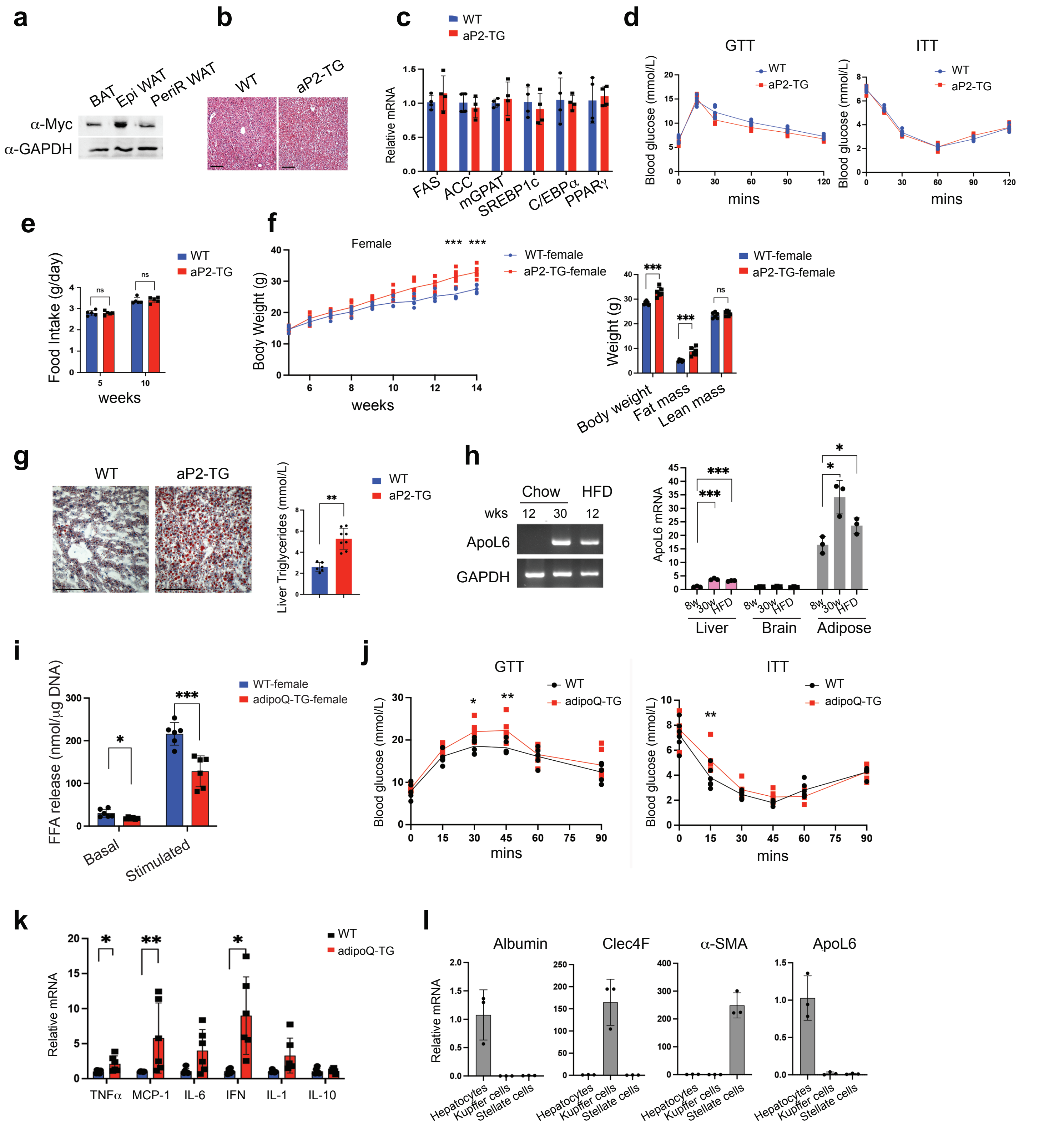

a
BAT
Epi WAT
PeriR WAT
α-Myc
α-GAPDH
b
WT
aP2-TG
c
WT
aP2-TG
Relative mRNA
FAS
ACC
mGPAT
SREBP1c
C/EBPα
PPARγ
d
GTT
ITT
Blood glucose (mmol/L)
mins
e
Food Intake (g/day)
weeks
f
Female
Body Weight (g)
weeks
WT-female
aP2-TG-female
Weight (g)
Body weight
Fat mass
Lean mass
g
Liver Triglycerides (mmol/L)
h
Chow
HFD
wks
ApoL6
GAPDH
ApoL6 mRNA
Liver
Brain
Adipose
i
FFA release (nmol/μg DNA)
WT-female
adipoQ-TG-female
Basal
Stimulated
j
GTT
ITT
WT
adipoQ-TG
Blood glucose (mmol/L)
mins
k
Relative mRNA
WT
adipoQ-TG
TNFα
MCP-1
IL-6
IFN
IL-1
IL-10
l
Albumin
Clec4F
α-SMA
ApoL6
Relative mRNA
Hepatocytes
Kupffer cells
Stellate cells

Supplementary Fig. 4. ApoL6 overexpression in female mice showed similar phenotype as males.

(**a**) Immunoblotting with Myc antibody for ApoL6-Myc protein levels in WAT and BAT from aP2-ApoL6 TG mice. (**b**) H&E staining of livers from 20 wk-old mice on chow diet (bar=100μm). (**c**) RT-qPCR of adipogenic transcription factors and lipogenic enzymes in WAT (n=4). Data represent mean ± SD. (**d**) GTT and ITT of mice on chow diet (*n*=6). (**e**) Food intake for HFD (*n*=6). (**f**) Female mice BW during HFD feeding and EchoMRI after HFD (*n*=6, ****p*<0.001). (**g**) Oil Red O staining (bar=100μm) and TAG levels in livers of mice on HFD (*n*=6, ***p*=0.0017). Data represent mean ± SD. (**h**) RT-PCR for ApoL6 expression in livers. RT-qPCR for ApoL6 expression in liver, brain, and WAT (*n*=3, ****p*<0.001, **p*=0.0115 and 0.044). (**i**) FFA release from dispersed adipocytes isolated from Female adipoQ-ApoL6 TG WAT (*n*=6, **p*=0.0118, ****p*<0.001). Data represent mean ± SD. (**j**) adipoQ-ApoL6 TG mice GTT (*n*=5, **p*=0.038, ***p*=0.0078) and ITT (*n*=5, ***p*=0.0092, two-way ANOVA) after HFD. Data represent mean ± SD. (**k**) RT-qPCR for inflammatory markers in WAT after HFD, showing more expression in adipoQ-ApoL6 TG than WT (*n*=6, **p*=0.018 and 0.016, ***p*=0.0038). (**l**) Hepatocytes, Kupffer cells, and stellate cells were isolated from livers of WT mice on HFD by pronase-collagenase perfusion and subsequent density-gradient centrifugation (Mederacke et al. 2015), (Mendoza et al. 2022). RT-qPCR for expression of albumin for hepatocytes, Clec4F for Kupffer cells, and α-SMA for stellate cells, as well as for ApoL6 expression (*n*=3). ApoL6 expression was detected in hepatocytes of mice after HFD.

References:

Mederacke, I., D. H. Dapito, S. Affo, H. Uchinami, and R. F. Schwabe. 2015. 'High-yield and high-purity isolation of hepatic stellate cells from normal and fibrotic mouse livers', *Nat Protoc*, 10: 305-15.

Mendoza, R., I. Banerjee, S. C. Reghupaty, R. Yetirajam, D. Manna, and D. Sarkar. 2022. 'Isolation and Culture of Mouse Hepatocytes and Kupffer Cells (KCs)', *Methods Mol Biol*, 2455: 73-84.

SFigure 5

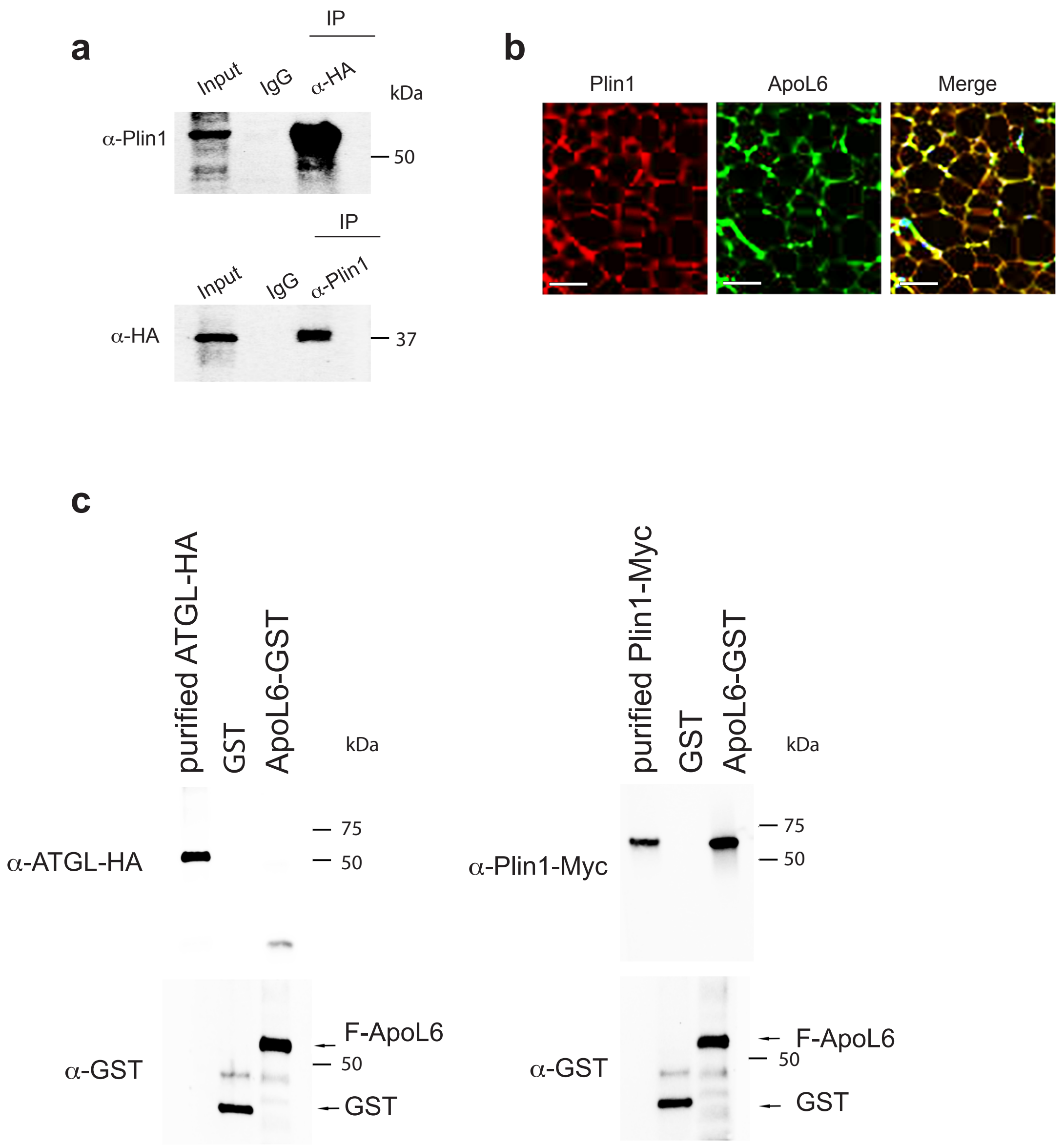
a
IP
Input
IgG
α-HA
kDa
α-Plin1
50
IP
Input
IgG
α-Plin1
α-HA
37
b
Plin1
ApoL6
Merge
c
purified ATGL-HA
GST
ApoL6-GST
kDa
75
α-ATGL-HA
50
α-GST
F-ApoL6
50
GST
purified Plin1-Myc
GST
ApoL6-GST
kDa
75
α-Plin1-Myc
50
α-GST
F-ApoL6
50
GST

Supplementary Fig. 5. ApoL6 interacts with Plin1 in human cells.
(**a**) Co-transfection of hApoL6-HA with Plin1 in HEK293 cells. Immunoblotting after lysates were IP with HA antibody and blotted with Plin1 antibody (upper). IP with Plin1 antibody and blotted with HA antibody (lower). (**b**) Immunofluorescence imaging of whole mount WAT and immunoblotting with Plin1 (mouse) and ApoL6 (rabbit) antibodies followed by Alexa fluor 594 anti-mouse and Alexa fluor 488 anti-rabbit antibodies, respectively (bar=10μm). (**c**) Purified ATGL-HA and Plin1-Myc were either incubated with GST or F-ApoL6-GST for overnight at 4°C. Immunoblotting after GST pull-down showing ApoL6-ATGL interaction was barely detectable.

SFigure 6

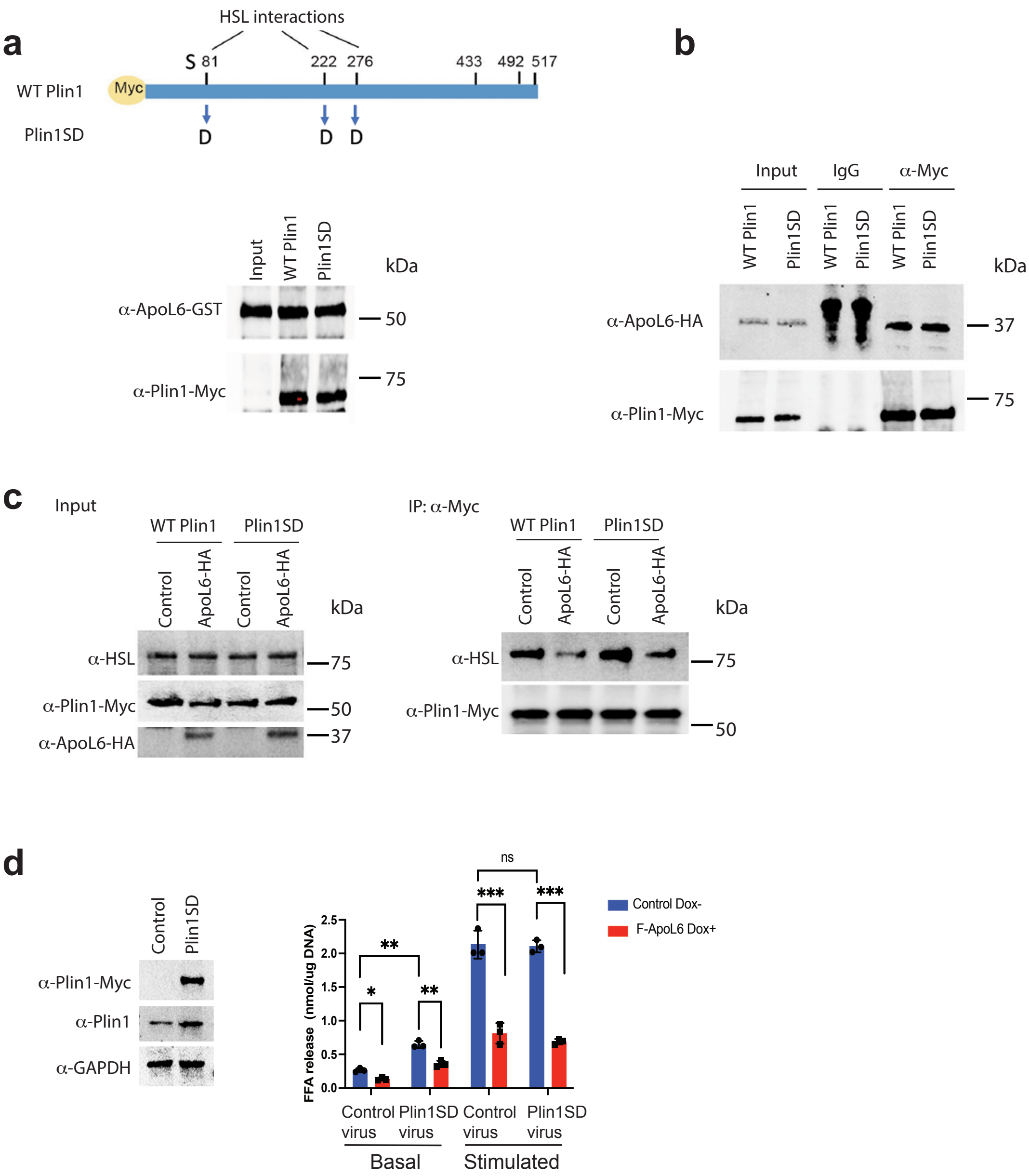
a
HSL interactions
S 81
222 276
433 492 517
WT Plin1
Myc
Plin1SD
D
D D
Input
WT Plin1
Plin1SD
kDa
α-ApoL6-GST
50
α-Plin1-Myc
75
b
Input
IgG
α-Myc
WT Plin1
Plin1SD
WT Plin1
Plin1SD
WT Plin1
Plin1SD
kDa
α-ApoL6-HA
37
α-Plin1-Myc
75
c
Input
WT Plin1
Plin1SD
Control
ApoL6-HA
Control
ApoL6-HA
kDa
α-HSL
75
α-Plin1-Myc
50
α-ApoL6-HA
37
IP: α-Myc
WT Plin1
Plin1SD
Control
ApoL6-HA
Control
ApoL6-HA
kDa
α-HSL
75
α-Plin1-Myc
50
d
Control
Plin1SD
α-Plin1-Myc
α-Plin1
α-GAPDH
FFA release (nmol/ug DNA)
2.5
2.0
1.5
1.0
0.5
0.0
ns
***
***
**
*
**
Control virus
Plin1SD virus
Control virus
Plin1SD virus
Basal
Stimulated
Control Dox-
F-ApoL6 Dox+

Supplementary Fig. 6. ApoL6 inhibits lipolysis regardless of the phosphorylation status of Plin1.

(**a**) S81, S222, S276 of Plin1 were all mutated to aspartic acid (Plin1SD). Purified WT Plin1 and Plin1SD were incubated with purified ApoL6-GST. Immunoblotting with GST antibody after IP with Myc antibody (left). (**b**) Co-transfection of ApoL6-HA and Myc tagged WT Plin1 and Plin1SD into 293FT cells. Immunoblotting with HA antibody after IP with Myc antibody. (**c**) Co-transfection of ApoL6-HA, HSL, and Myc tagged either WT Plin1 or Plin1SD into HEK293FT cells (left). Immunoblotting with HSL antibody after IP with Myc antibody (right). These results indicate that ApoL6 prevents Plin1-HSL interaction regardless of Plin1 phosphorylation status. (**d**) Immunoblotting of lysates of differentiated 3T3-L1 adipocytes infected with control lentivirus or Plin1SD lentivirus along with inducible ApoL6-HA lentivirus (left). FFA release was measured (right) in basal and isoproterenol treated conditions (n=3, *p=0.0211, **p=0.0023 and 0.0028, ***p<0.001). Data represent mean ± SD. Experiments were repeated twice.
