# Supplementary material for: ApoL6 associates with lipid droplets and disrupts Perilipin1-HSL interaction to inhibit lipolysis
Source: Nat Commun. 2024 Jan 2;15:186. doi: 10.1038/s41467-023-44559-3 (PMC10762002; doi:10.1038/s41467-023-44559-3)
Supplement: Supplementary file 2 — Reporting Summary [file 41467_2023_44559_MOESM2_ESM.pdf]

nature portfolio

Corresponding author(s): NCOMMS-22-43675-T
Last updated by author(s): 12-14-2022

Reporting Summary

Nature Portfolio wishes to improve the reproducibility of the work that we publish. This form provides structure for consistency and transparency in reporting. For further information on Nature Portfolio policies, see our Editorial Policies and the Editorial Policy Checklist.

Statistics

For all statistical analyses, confirm that the following items are present in the figure legend, table legend, main text, or Methods section.

n/a	Confirmed	
☐	☒	The exact sample size (*n*) for each experimental group/condition, given as a discrete number and unit of measurement
☐	☒	A statement on whether measurements were taken from distinct samples or whether the same sample was measured repeatedly
☐	☒	The statistical test(s) used AND whether they are one- or two-sided<br>*Only common tests should be described solely by name; describe more complex techniques in the Methods section.*
☒	☐	A description of all covariates tested
☐	☒	A description of any assumptions or corrections, such as tests of normality and adjustment for multiple comparisons
☐	☒	A full description of the statistical parameters including central tendency (e.g. means) or other basic estimates (e.g. regression coefficient) AND variation (e.g. standard deviation) or associated estimates of uncertainty (e.g. confidence intervals)
☒	☐	For null hypothesis testing, the test statistic (e.g. *F*, *t*, *r*) with confidence intervals, effect sizes, degrees of freedom and *P* value noted<br>*Give P values as exact values whenever suitable.*
☒	☐	For Bayesian analysis, information on the choice of priors and Markov chain Monte Carlo settings
☒	☐	For hierarchical and complex designs, identification of the appropriate level for tests and full reporting of outcomes
☒	☐	Estimates of effect sizes (e.g. Cohen's *d*, Pearson's *r*), indicating how they were calculated

Our web collection on statistics for biologists contains articles on many of the points above.

Software and code

Policy information about availability of computer code

Data collection	N/A
Data analysis	Microsoft Excel, Prism8, ImageJ1.52

For manuscripts utilizing custom algorithms or software that are central to the research but not yet described in published literature, software must be made available to editors and reviewers. We strongly encourage code deposition in a community repository (e.g. GitHub). See the Nature Portfolio guidelines for submitting code & software for further information.

Data

Policy information about availability of data

All manuscripts must include a data availability statement. This statement should provide the following information, where applicable:

- Accession codes, unique identifiers, or web links for publicly available datasets
- A description of any restrictions on data availability
- For clinical datasets or third party data, please ensure that the statement adheres to our policy

Provide your data availability statement here.

Human research participants

Policy information about studies involving human research participants and Sex and Gender in Research.

Reporting on sex and gender	N/A
Population characteristics	N/A
Recruitment	N/A
Ethics oversight	N/A

Note that full information on the approval of the study protocol must also be provided in the manuscript.

Field-specific reporting

Please select the one below that is the best fit for your research. If you are not sure, read the appropriate sections before making your selection.

☒ Life sciences ☐ Behavioural & social sciences ☐ Ecological, evolutionary & environmental sciences

For a reference copy of the document with all sections, see nature.com/documents/nr-reporting-summary-flat.pdf

Life sciences study design

All studies must disclose on these points even when the disclosure is negative.

Sample size	The sample size was large enough to determine statistically significant effects and was determined based upon other studies with similar methodologies (PMID: 32034158).
Data exclusions	No data were excluded from analysis.
Replication	Experiments were repeated independently at least 2-3 times and successfully reproducible.
Randomization	Mice (age and sex matched) and cells were randomly assigned to different treatment groups.
Blinding	Blind test was performed for data collection and analysis.

Reporting for specific materials, systems and methods

We require information from authors about some types of materials, experimental systems and methods used in many studies. Here, indicate whether each material, system or method listed is relevant to your study. If you are not sure if a list item applies to your research, read the appropriate section before selecting a response.

Materials & experimental systems

n/a	Involved in the study	
☐	☒	Antibodies
☐	☒	Eukaryotic cell lines
☒	☐	Palaeontology and archaeology
☐	☒	Animals and other organisms
☒	☐	Clinical data
☒	☐	Dual use research of concern

Methods

n/a	Involved in the study	
☒	☐	ChIP-seq
☒	☐	Flow cytometry
☒	☐	MRI-based neuroimaging

Antibodies

Antibodies used	ApoL6 Rabbit Ab lab generated<br>perilipin goat Ab abcam ab61682<br>perilipin Rabbit Ab Cell Signaling 9349s<br>ATGL Rabbit Ab Cell Signaling 2138s<br>HSL Ab Cell Signaling 4107s

CGI58 Ab Santa Cruz Bio sc-102285
C/EBPa Ab Santa Cruz Bio sc-61x
PPARg Ab Santa Cruz Bio sc-7273x
GAPDH Ab Santa Cruz Bio sc-47724
ApoL6 Rabbit Ab lab generated
perilipin goat Ab abcam ab61682
perilipin Rabbit Ab Cell Signaling 9349s
ATGL Rabbit Ab Cell Signaling 2138s
HSL Ab Cell Signaling 4107s
CGI58 Ab Santa Cruz Bio sc-102285
C/EBPa Ab Santa Cruz Bio sc-61x
PPARg Ab Santa Cruz Bio sc-7273x
GAPDH Ab Santa Cruz Bio sc-47724

Validation

The validation statements of commercial antibodies were validated using 293FT lysates overexpressing cDNAs

Eukaryotic cell lines

Policy information about cell lines and Sex and Gender in Research

Cell line source(s)

HEK293T(CRL-1573) , 3T3L1 cells, primary human subcutaenous fibroblasts were obtained from ATCC.

Authentication

Both cell lines were authenciated by lipid accumulation during adipogenesis by RT-qPCR analysis.

Mycoplasma contamination

Cells were tested to be negative for mycoplasma contamination by PCR screening.

Commonly misidentified lines
(See ICLAC register)

None

Animals and other research organisms

Policy information about studies involving animals; ARRIVE guidelines recommended for reporting animal research, and Sex and Gender in Research

Laboratory animals

8~25 weeks old C57BL/6 wild type mice were used. aP2-ApoL6 TG, Adiponectin-ApoL6 TG, Crispr ApoL6 KO were backcrossed tp C57BL/6 more than 4 generations. Animals were housed at 23C. TG and KO were compared to their wildtype littermates.

Wild animals

N/A

Reporting on sex

We performed experiments with both males and females. We here only presented data obtained from males. When we performed some experiments (body weight, tissue distribution etc) with females, we obtained similar results; There was no difference in sex.

Field-collected samples

N/A

Ethics oversight

All the methods involving live mice were carried out in accordance with the approve guidelines. All experiments protocols were approved by the Animal Care and Use Committee (AUP-2016-11-9317-2) at UC Berkeley.

Note that full information on the approval of the study protocol must also be provided in the manuscript.
